# Comparative and Evolutionary Analysis of Grass Pollen Allergens Using *Brachypodium distachyon* as a Model System

**DOI:** 10.1371/journal.pone.0169686

**Published:** 2017-01-19

**Authors:** Akanksha Sharma, Niharika Sharma, Prem Bhalla, Mohan Singh

**Affiliations:** 1 Plant Molecular Biology and Biotechnology Laboratory, Faculty of Veterinary and Agricultural Sciences, University of Melbourne, Parkville, Melbourne, VIC, Australia; 2 Australian Centre for Plant Functional Genomics (ACPFG), School of Agriculture, Food and Wine, University of Adelaide, Waite Campus, Plant Genomics Centre, Hartley Grove, Urrbrae, SA, Australia; Oklahoma State University, UNITED STATES

## Abstract

Comparative genomics have facilitated the mining of biological information from a genome sequence, through the detection of similarities and differences with genomes of closely or more distantly related species. By using such comparative approaches, knowledge can be transferred from the model to non-model organisms and insights can be gained in the structural and evolutionary patterns of specific genes. In the absence of sequenced genomes for allergenic grasses, this study was aimed at understanding the structure, organisation and expression profiles of grass pollen allergens using the genomic data from *Brachypodium distachyon* as it is phylogenetically related to the allergenic grasses. Combining genomic data with the anther RNA-Seq dataset revealed 24 pollen allergen genes belonging to eight allergen groups mapping on the five chromosomes in *B*. *distachyon*. High levels of anther-specific expression profiles were observed for the 24 identified putative allergen-encoding genes in Brachypodium. The genomic evidence suggests that gene encoding the group 5 allergen, the most potent trigger of hay fever and allergic asthma originated as a pollen specific orphan gene in a common grass ancestor of Brachypodium and Triticiae clades. Gene structure analysis showed that the putative allergen-encoding genes in Brachypodium either lack or contain reduced number of introns. Promoter analysis of the identified Brachypodium genes revealed the presence of specific *cis*-regulatory sequences likely responsible for high anther/pollen-specific expression. With the identification of putative allergen-encoding genes in Brachypodium, this study has also described some important plant gene families (e.g. expansin superfamily, EF-Hand family, profilins etc) for the first time in the model plant Brachypodium. Altogether, the present study provides new insights into structural characterization and evolution of pollen allergens and will further serve as a base for their functional characterization in related grass species.

## Introduction

Grass pollens constitute a major worldwide source of airborne allergens and are known to be responsible for triggering allergic rhinitis and exacerbating asthma [1]. Grasses belong to the family Poaceae (Gramineae) and are grouped with the sedges, rushes, and other monocots belonging to the order Poales. The family Poaceae is the fourth largest family of flowering plants with more than 600 genera and 10,000 species. The three important subfamilies of Poaceae, namely the subtropical Chloridoideae and Panicoideae, and the temperate Pooideae are known to constitute the major bulk of allergenic pollen [2, 3]. *Cynodon dactylon* (Bermuda grass), a popular southern turf grass, is recognized as the chief allergenic grass species of Chloridoideae [2–4]. Likewise, the Panicoideae subfamily mainly includes *Sorghum halepense* (Johnson grass) and *Paspalum notatum* (Bahia grass) as significant allergenic members [2–5]. Notably, the temperate Pooideae subfamily represents the majority and pivotal allergenic grasses such as *Lolium perenne* (ryegrass), *Phleum pratense* (timothy grass), *Holcus lanatus* (velvet grass), *Phalaris aquatica* (canary grass), *Poa pratensis* (bluegrass) and *Dactylis glomerata* (orchard grass) [2, 3]. In the recent years, a member of the same subfamily (Pooideae), *Brachypodium distachyon* (Brachypodium) has been recognised as a model plant for structural and functional genomics research in grasses. It is the first member to be sequenced within the Pooideae subfamily and shares a high degree of synteny with other grasses.

Grass pollen allergenicity can be attributed to certain proteins which are rapidly released from the pollen grain upon hydration [1, 6]. These proteins possess multiple IgE-reactive epitopes that can bind cross-link adjacent IgE molecules leading to degranulation of mast cells leading to clinical symptoms of allergy. Allergens (proteins) identified in one species often have homologues in other evolutionary related species, exhibiting similar physicochemical and immunological properties [1, 7]. The conserved IgE epitopes on these homologous proteins lead to allergenic cross-reactivity, implying that allergic individuals sensitised to an allergen can show allergenic reactivity to related allergen from a different species.

Pollen allergens found in different grass species which exhibit structural similarities and allergic cross-reactivities have been classified as group 1, 2, 3, 4, 5, 6, 7, 11, 12, 13, 22, 24 allergens (Table 1) [8–19]. Out of these, group 1 and group 5 allergens were found to be the most clinically relevant, since 95 and 65–85% of patients, respectively, are reported to be sensitised to them [1, 4]. Together group 1 and five allergens account for more than 80% of total IgE reactivity of grass pollen proteins.

**Table 1:** Overview of characteristics of grass pollen allergens [8–19].

Allergen	Allergen nature and characteristics
Group 1 (31–35 kDa)	Acidic glycoproteins, exist in multiple isoforms distinguished by their respective isoelectric points (pIs), apparent molecular weights and amino acid sequences, belong to a subfamily of structurally related proteins called β-expansins, which are cell-wall loosening proteins.
Group 2/3 (10–12 kDa)	Group 2 (acidic) and group 3 (basic) are non-glycosylated proteins, share a high degree of sequence homology (40% identity, 60–70% similarity) with the C-terminal part of group 1 allergens that suggest a common origin by gene duplication.
Group 4 (50–60 kDa)	High molecular weight basic glycoproteins, sequence similarity to the berberine bridge enzymes (BBE), a member of a flavoprotein oxidoreductase superfamily.
Group 5 (27–33 kDa)	A heterogenous group of non-glycosylated proteins with multiple isoforms varying in pIs and primary sequences, examined in only Pooideae subfamily of grasses and antibodies raised against group 5 allergens failed to detect cross-reactive antigens outside of the subfamily.
Group 6 (11 kDa)	Acidic, non-glycosylated proteins, so far been detected in Timothy grass pollen extracts only.
Group 7 (8–12 kDa)	Small proteins belonging to the polcalcin family of calcium-binding proteins with homologues present in pollen of all monocots and dicots.
Group 11 (16–20 kDa)	Glycoproteins, belonging to the soybean-trypsin-inhibitor-like family, structurally similar to pollen allergens from the olive tree (Ole e 1).
Group 12 (14 kDa)	Members of the conserved profilin protein family present in all eukaryotic cells.
Group 13 (50–60 kDa)	Members of a polygalacturonase like protein family.
Group 22 (9 kDa)	Enolase, so far only identified in Bermuda grass pollen extract.
Group 24 (21 kDa)	Pathogenesis-related protein (PR-1), so far only identified in Bermuda grass pollen extract.

The distribution of allergens across the different grasses varies: some allergens are shared across the entire plant kingdom (e.g. group 12) or between pollen plants (e.g. group 7). Somes allergens are restricted to Poaceae (group 1) or Pooideae (group 5). Group 4, 7, 11 and 12 allergens are not grass-specific while group 12 allergen (profilin) is present in all eukaryotic cells. However, no comprehensive study focussing on origin and evolution of pollen allergen families in the genome of allergenic grass species is available to date. Availability of fully sequenced genome for pooid grasses such as ryegrass and timothy grass can significantly facilitate addressing questions such as allergen sequence homology and evolutionary diversity of the isoforms.

The recent availability of the genomic sequence of *Brachypodium distachyon* [20–24] has provided a powerful genomic resource to further our understanding grass pollen allergens repertoire. The availability of protocols for *Brachypodium* transformation will further allow addressing of allergen-encoding gene function through RNAi or gene editing technologies (CRISPR). Also, because of the phylogenetic relatedness and extensive synteny shared among grass genomes, the genetic information from Brachypodium would be beneficial for understanding the structure, organisation and evolution of pollen allergens in clinically significant grass genera.

Thus in the present study, an extensive genome-wide analysis was performed to (i) identify putative allergen-encoding genes in *B*. *distachyon* genome, (ii) analyze gene structures and protein domains, (iii) examine the phylogenetic and evolutionary relationships between *B*. *distachyon* putative allergens and other related grasses, (iv) identify potential promoters targeting pollen allergen genes and (v) investigate the expression patterns of putative allergen-encoding genes in Brachypodium using available RNA-Seq data.

## Materials and Methods

### Sequence retrieval and identification of conserved domains in different groups of pollen allergens

A complete set of allergens sequences has been reported from ryegrass (*Lolium perenne* L.) and Timothy grass (*Phleum pratense* L.) [1, 4]. Therefore, protein sequences from these two grass species were selected as representative members and were later used as seed sequences to search against *B*. *distachyon* proteome database. Lol p 1 (P14946) for group 1, Lol p 2 (P14947) for group 2/3, Lol p 4 (Q5TIW3) for group 4, Lol p 5 (Q40240) for group 5, Phl p 7 (O82040) for group 7, Lol p 11 (Q7M1X5) for group 11, Phl p 12 (P35079) for group 12 and Phl p 13 (CAB42886) for group 13 were chosen as representative sequences. All the protein sequences were retrieved from NCBI (http://www.ncbi.nlm.nih.gov/) were listed in S1 Table. Conserved domains were identified in each pollen allergen group using SDAP (Structural database of allergenic proteins; http://fermi.utmb.edu/SDAP) and further verified using SMART (Simple Modular Architecture Research Tool) (http://smart.embl-heidelberg.de) and Pfam (http://pfam.sanger.ac.uk/). Schematic diagrams of pollen allergens belonging to different groups with conserved domains were constructed using DOG 2.0 (Domain Graph, version 2.0) (http://dog.biocuckoo.org/) [25].

### Identification of putative allergen-encoding genes in *B*. *distachyon*

Representative pollen allergen sequences of ryegrass (*Lolium perenne* L.) and Timothy grass (*Phleum pratense* L.) were searched against *B*. *distachyon* proteome database (v2.1 available at Phytozome (v10.1, http://www.phytozome.net/)) using Basic Local Alignment Search Tool algorithms (BLASTP) under default parameters. The resultant top hits from BLASTP searches of *B*. *distachyon* were analysed for the presence of exemplar conserved domains using SMART and Pfam. The presence of signal peptides was predicted by Signal P4.1 (http://www.cbs.dtu.dk/services/SignalP/) and N-glycosylation sites by NetNGlyc 1.0 (http://www.cbs.dtu.dk/services/NetNGlyc/).

### Multiple sequence alignment and phylogenetic analysis

Protein sequences of *B*. *distachyon*, *Lolium perenne* and *Phleum pratense* retrieved above were aligned using the multiple sequence alignment (MSA) tool CLUSTALX2.0, using Gonnet Protein Weight Matrix with default parameters and phylogenetic trees were constructed by using neighbour-joining (NJ) method. The stability of nodes was tested by bootstrap analysis with 100 replicates and trees were viewed using MEGA v6.0 [26].

### Chromosomal location and gene structure

The information about the chromosomal location of allergen-encoding genes, i.e. chromosome number, start position, end position and gene orientation in Brachypodium was retrieved from the *B*. *distachyon* genome information file (v2.1). This information was used to locate the genes on a schematic map. Further, the exon-intron organisation of these genes was deduced using GSDS (Gene structure display server; http://gsds.cbi.pku.edu.cn/) via alignment of the cDNAs with their corresponding genomic DNA sequences [27].

### Promoter analysis and homology modelling

The upstream DNA sequences of putative allergen-encoding genes were obtained from the *B*. *distachyon* genome dataset (v2.1) for locating known *cis*-regulatory elements (CREs) using SIGNALSCAN program available in Plant *cis*-Regulatory DNA elements (PLACE) (http://www.dnd.affrc.go.jp/PLACE/) database [28]. Further, protein structure determination by homology modelling was performed using Phyre2 (Protein Homology/AnalogY Recognition Engine V 2.0; http://www.sbg.bio.ic.uk/phyre2) under ‘intensive’ mode [29].

### Expression profiling using published RNA-Seq dataset

To elucidate the expression profiles of putative allergen-encoding genes in *B*. *distachyon*, Illumina RNAseq reads from 9 distinct tissues including immature leaves, pre-and post-emergence flowers, anther, pistil, whole seed at 5 DAP (days after pollination), whole seed at 10 DAP, embryo at 25 DAP and endosperm at 25 DAP, were downloaded from the Sequence Read Archive (SRA) at the National Centre for Biotechnology Information (SRP008505) [30]. RNA-Seq reads in the fastq format were then used for mapping against the latest version of *B*. *distachyon* transcripts (v2.1) using RSEM v.1.2.19 and Bowtie v0.12.8 under default settings [31]. RSEM outputs abundance estimates and transcripts expression levels were given as FPKM (fragments per kilobase of exon per million mapped fragments) values. The FPKM values of each transcript from replicate samples of immature leaves and embryo at 25 DAP were averaged to obtain the final value and transcripts with FPKM greater than or equal to one were considered as expressed. Heat maps were generated to represent the gene expression ratio in percentage with FPKM value shown in the most highly expressed sample. To visualise the gene expression clusters among homologues and /or paralogs, heat maps were plotted against the phylogenetic tree constructed using *B*. *distachyon* protein sequences.

## Results

### Identification of conserved domains in pollen allergen groups

**Fig 1:**
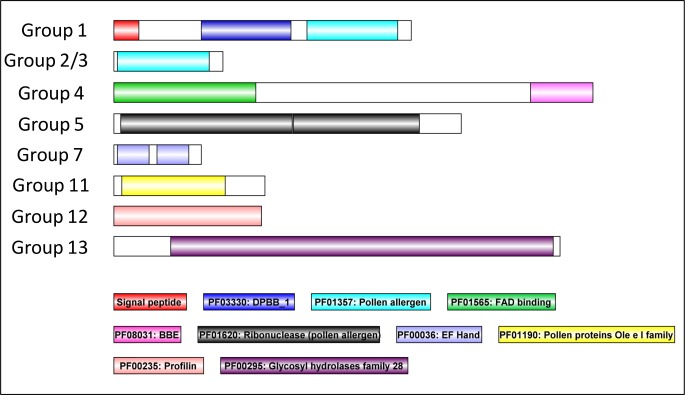
Protein domain architecture of representative allergen proteins from each group. The protein domains in pollen allergen groups are identified by SDAP (Structural Database of Allergenic Proteins) database and verified using the SMART (http://smart.embl-heidelberg.de) and PFAM (http://pfam.sanger.ac.uk/). Lol p 1 (P14946) for group 1, Lol p 2 (P14947) for group 2/3, Lol p 4 (Q5TIW3) for group 4, Lol p 5 (Q40240) for group 5, Phl p 7 (O82040) for group 7, Lol p 11 (Q7M1X5) for group 11, Phl p 12 (P35079) for group 12 and Phl p 13 (CAB42886) for group 13 were chosen as representative members respectively.

Ryegrass and Timothy grass protein sequences belonging to eight different allergen groups were chosen as representative members and the conserved domains within each group of pollen allergens were identified. Fig 1 shows the presence of different conserved domains within each group of pollen allergens. This information was utilised for identifying allergen homologs in *B*. *distachyon* by presence of conserved domains. Group 1 allergens are characterised with a Rare lipoprotein-A (RlpA)-like double-psi beta-barrel (DPBB_1) domain and a pollen allergen domain while group 2/3 allergens contain a single pollen allergen domain. Group 4 allergens include both FAD binding and BBE domains. Group 5 allergens encode two ribonuclease (pollen allergen) domains. Grass pollen proteins of group 7 are characterised by two EF-Hand domains and group 11 with an Ole e one domain respectively. Similarly, Group 12 allergens include a profilin domain while group 13 allergens contain glycosyl hydrolases family 28 domain.

### Identification, characterization and expression profiles of putative homologs of pollen-allergen genes in *B*. *distachyon*

**Fig 2:**
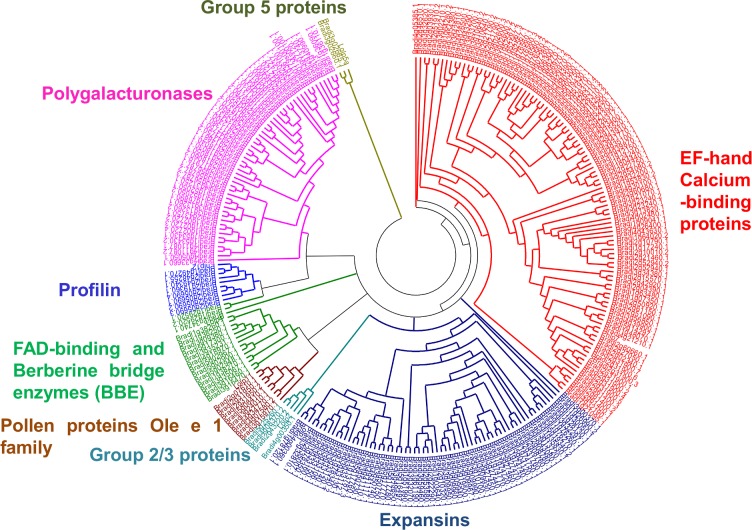
Phylogenetic analysis of pollen allergen protein families in *B*. *distachyon* and representative member of each group from Ryegrass and Timothy grass. The protein sequences were aligned by Clustal X2.0 and unrooted phylogenetic tree was constructed by neighbour-joining method with 100 bootstrap replicates. Branches with less than 50% bootstrap support were collapsed. The tree was divided into eight phylogenetic clusters. The members were distinctly coloured to represent respective protein family. Lol p 1 (P14946) for group 1, Lol p 2 (P14947) for group 2/3, Lol p 4 (Q5TIW3) for group 4, Lol p 5a (Q40240) for group 5, Phl p 7 (O82040) for group 7, Lol p 11 (Q7M1X5) for group 11, Phl p 12 (P35079) for group 12 and Phl p 13 (CAB42886) for group 13 were chosen as representative members respectively.

A total of 257 Brachypodium proteins were identified using ryegrass and timothy grass representative allergen protein sequences as queries against Brachypodium genome dataset. All the identified 257 proteins were then analysed to confirm the presence of conserved protein domains as identified earlier in different groups of pollen allergens. The phylogenetic relatedness of the identified 257 Brachypodium proteins was further examined by MSA and a Neighbour-Joining phylogenetic tree (Fig 2). The proteins were categorised into eight different groups or protein families (Expansins, group 2/3 allergens, FAD-binding and berberine bridge enzymes, group 5 allergens, EF-hand calcium binding proteins, pollen proteins Ole e one like profilins and polygalacturonases) indicating that all the groups of grass pollen allergens are represented in *B*. *distachyon* genome.

#### Expansin protein family in *B*. *distachyon* and Group 1 allergens

In total, 64 proteins were identified as Expansins by BlastP search of Lol p 1 (P14946, ryegrass group 1 allergen) as a query against Brachypodium proteome database. S1 Fig shows phylogenetic relationships, exon/intron structure, domain architecture and expression profile of the expansin protein family in *B*. *distachyon*. The proteins were further categorised into three different sub-classes of expansins (β-expansins, expansin-like and α-expansins) by their functional annotations. The largest subgroup was categorised as α-expansins, with 32 members as shown in clade 3 (green shade). The second largest subgroup was β-expansins, containing 26 members clade 1 (red shade). The smallest subgroup was expansin-like proteins that included six members in clade-2 (blue shade). As group 1 allergens belong to the family of β-expansins, we identified four Brachypodium proteins (Bradi1g78120.1, Bradi4g00360.1, Bradi3g32810.1 and Bradi2g35007.1) as putative group 1 allergens, closely clustered with Lol p 1 and exhibited anther-specific expression as compared to other vegetatively expressed β-expansins. Protein sequence identities of Brachypodium group 1 isoforms with known group 1 allergens from other grasses range between 53% and 89% (S2 Table). Bradi1g78120.1 bears closest sequence relationship in the grasses to a Velvet grass group 1 allergen (Hol l 1) showing 100% sequence coverage and 82% sequence identity. To appropriately identify group 1 allergen isoforms, MSA was carried out for four identified group 1 isoforms in Brachypodium and known group 1 proteins from other allergenic grasses (S2 Fig). Alignment results highlighted the conservation of typical structural characteristics of group 1 allergens [1] in all four isoforms of Brachypodium that included an N-glycosylation site at amino acid position 9, hydroxyproline residues at positions 5 and 8 and seven conserved cysteine residues. Sequence alignments also showed the conservation of IgE-binding residues in Group 1 allergens and Brachypodium homologs (S2 Fig) [32]. Thus, we conclude, four Brachypodium transcripts being transcribed by putative group 1 allergen-encoding genes (S1 Fig).

#### Group 2 allergens

We identified four proteins (Bradi5g25260.1, Bradi4g08370.2, Bradi2g43220.2 and Bradi4g00350.1) as group 2/3 allergens by protein domain architecture and anther-specific expression profiles of their corresponding transcripts (S3 Fig). S4 Fig illustrates the sequence alignments of the four identified group 2/3 homologs in Brachypodium with other known group 2/3 allergens. A previous study in Timothy grass defined the three-dimensional (3D) structure of a complex between Phl p 2 and a Phl p 2-specific Fab derived from an IgE antibody. The IgE-binding epitope comprised nine critical residues grouped into four sequentially distant segments of the allergen molecule [33]. We observed that all the nine purportedly critical residues were conserved in three Brachypodium proteins (Bradi5g25260.1, Bradi4g08370.2 and Bradi2g43220.2) while Bradi4g00350.1 exhibited seven out of nine epitope critical residues (S4 Fig). Protein sequence identities of Brachypodium group 2/3 isoforms with group 2 allergens range between 53% and 66% (S2 Table).

#### FAD-binding/Berberine Bridge Enzymes (BBE) protein family in *B*. *distachyon* and Group 4 allergens

BlastP search with Lol p 4 sequence identified 16 proteins in *B*. *distachyon* proteome that belong to a family of FAD binding and berberine bridge enzymes (BBE) proteins by the presence of conserved Pfam domains (PF01565 and PF08031) that also included group 4 allergens. S5 Fig shows their phylogenetic relationships, exon/intron structure, domain architecture and their transcript expression profiles. Since group 4 allergens exhibited sequence similarity to FAD binding and/or berberine bridge enzymes (BBE) proteins, we found only one isoform of group 4 in Brachypodium (Bradi1g38260.1) whose corresponding transcript showed anther- specific expression pattern and was grouped closely with Lol p 4 in the phylogenetic tree (S5 Fig). S6 Fig shows MSA of Bradi1g38260.1 and group 4 proteins from other grasses. The results displayed the presence of N-glycosylation sites and conserved residues among group 4 proteins. Bradi1g38260.1 protein sequence showed 99% sequence coverage and 81% sequence identity to Sec c 4 and Tri a 4 allergens respectively (S2 Table).

#### Group 5 allergens

Using ryegrass group 5 allergen (Lol p 5; Q40240) as a query against Brachypodium proteome dataset, only three Brachypodium proteins (Bradi3g02660.1, Bradi3g02665.1 and Bradi2g48255.1) were revealed to possess conserved group 5 allergen protein domains (PF01620). Further, expression profiles of the corresponding transcripts also revealed high anther-specific expression pattern (S7 Fig). S8 Fig exhibits MSA of the identified group 5 isoforms in Brachypodium with known group 5 proteins from other allergenic grasses of the Pooideae subfamily. The alignments revealed the presence of conserved IgE-binding residues [34] among different group 5 proteins and Brachypodium homologs (S7 Fig). Protein sequence identities of Brachypodium group 5 isoforms with other known group 5 allergens vary greatly (from 43% to 77%) as typical of sequence variation in group 5 allergens (S2 Table).

#### Calcium-binding EF-hand family proteins and group 7 allergens

BlastP search of Timothy grass group 7 allergen (Phl p 7; O82040) against *B*. *distachyon* proteome identified 100 proteins that contained at least one EF-hand domain (PF00036). (S9 Fig). Interestingly, only one protein (Bradi4g21210.1) clustered closely with Phl p 7 in the first clade of the phylogenetic tree and thus, identified as group 7 isoform in Brachypodium. Further, the expression profile of Bradi4g21210.1 confirmed anther specificity of this group 7 homolog. (S9 Fig). S10 Fig shows the sequence alignments of the identified group 7 homolog in Brachypodium with Timothy grass (Phl p 7; O82040) and Bermuda grass (Cyn d 7; P94092) group 7 proteins. The alignment results highlighted the presence of two conserved EF-hand domains in all the protein sequences. Bradi4g21210.1 showed 100% protein sequence coverage and 94% sequence identity with a Cyn d 7 pollen allergen (S2 Table).

#### Pollen proteins ole e 1 protein family and group 11 allergens

We identified 11 proteins belonging to Ole e 1 family based on the presence of protein domain (PF01190) as observed in ryegrass group 11protein (Lol p 11; Q7M1X5) (Fig 1). S11 Fig shows phylogenetic relationships, exon/intron structure, domain architecture and expression profile of Ole e 1 protein family in *B*. *distachyon*. Four proteins (Bradi2g07610.1, Bradi2g31950.1, Bradi2g31950.2 and Bradi5g08410.1), comprising the first clade of phylogenetic tree were grouped closely with Lol p 11 and their corresponding transcripts also exhibited anther-specific expression pattern. The four homologs thus are identified as group 11 proteins in Brachypodium. Sequence similarities of group 11 isoforms in *B*. *distachyon* with ryegrass (Lol p 11; Q7M1X5) and Timothy grass (Phl p 11; AAN32987) ranges between 45% and 88% (S2 Table). MSA of Lol p 11, Phl p 11 and Brachypodium group 11 isoforms marked the presence of conserved amino acid residues in group 11 allergens with a single N-linked glycosylation site at residue 24 and six conserved cysteine residues (S12 Fig).

#### Profilin protein family in *B*. *distachyon* and group 12 allergens

Group 12 allergens are the members of profilin protein family and BlastP result of Timothy grass group 12 allergen (Phl p 12; P35079) as queried against Brachypodium proteome identified seven proteins belonging to this family. We categorised two homologs (Bradi2g19360.1 and Bradi2g49340.1) that are grouped together with Phl p 12 in the first clade of the phylogenetic tree as group 12 proteins in *B*. *distachyon* by the clear demarcation of the anther-specific expression profile of the two corresponding transcripts (S13 Fig). S2 Table shows protein sequence coverage and identity of group 12 homologs from Brachypodium and other grasses. Sequence similarities of Brachypodium group 12 homologs with known group 12 proteins were observed with 100% coverage and 84–92% identity. S14 Fig shows the MSA of two identified group 12 homologs in Brachypodium with group 12 proteins from other grasses. The alignment highlighted the presence of cysteine residues at two conserved positions.

#### Polygalacturonases/Glycosyl hydrolases family 28 and group 13 allergens

Group 13 allergens belong to the family of pectin-degrading enzymes (i.e., polygalacturonases). BlastP analysis with Timothy grass group 13 allergen (Phl p 13; CAB42886) sequence as a query against Brachypodium proteome identified 52 homologs that contain the conserved Glycosyl hydrolases family 28 domain (PF00295) as observed in Phl p 13 (Fig 1). S15 Fig shows phylogenetic relationships, exon/intron structure, domain architecture and expression profile of polygalacturonases family in *B*. *distachyon*. On the basis of the anther-specific expression pattern of their corresponding transcripts, five candidate genes (Bradi1g36110.1, Bradi1g36090.1, Bradi1g40990.1, Bradi2g13740.1 and Bradi3g07120.1) were identified as encoding group 13 allergens in Brachypodium. MSA of five Brachypodium homologs and group 13 proteins from Bermuda grass and maize (Phl p 13, Zea m 13) suggested the conservation of polygalacturonase domains, 14 cysteine residues at conserved positions and N-glycosylation sites (S16 Fig). S2 Table shows protein sequence identities of Brachypodium group 13 homologs with Phl p 13 and Zea m 13 that range between 58% and 85%.

### Comparative analysis of pollen allergen homologs in *B*. *distachyon* and other allergenic grasses

**Fig 3:**
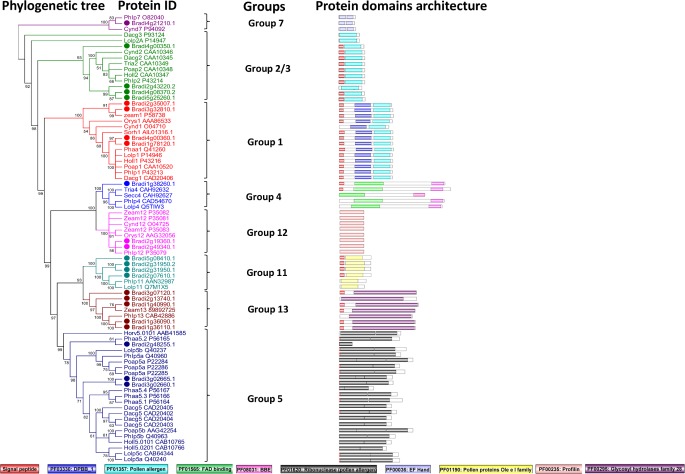
Phylogenetic tree and domain architecture of pollen allergens. Neighbour-Joining phylogenetic tree is constructed based on protein alignments of putative allergen-encoding proteins of *B*. *distachyon* and known pollen allergens from other grasses using ClustalX2. Bootstrap values greater than 50% are shown at the nodes. The schematic diagrams show the domain organization of these proteins as obtained from SMART and PFAM searches. Different domains are indicated by the use of different colours as shown at the bottom of the figure. The accession numbers for the sequences used in the alignment are also listed in the figure.

In total, we have identified 24 homologs of pollen allergens in *B*. *distachyon* belonging to eight different groups. To investigate the molecular evolution of pollen allergens from various allergenic grasses and their homologs in Brachypodium, we constructed a phylogenetic tree using the amino acid sequences (Fig 3). The sequences of all the proteins used are given in S1 Table. Analysis of the tree showed that group 4 and group 12 have clustered together but possessed different domains. A similar observation was made with group 11 and group 13 that were branched into a single clade, but both groups encoded different domains. This observation suggests a greater sequence divergence in some of the allergen groups during evolution.

### Expression profile of pollen allergen-encoding transcripts of *B*. *distachyon*

**Fig 4:**
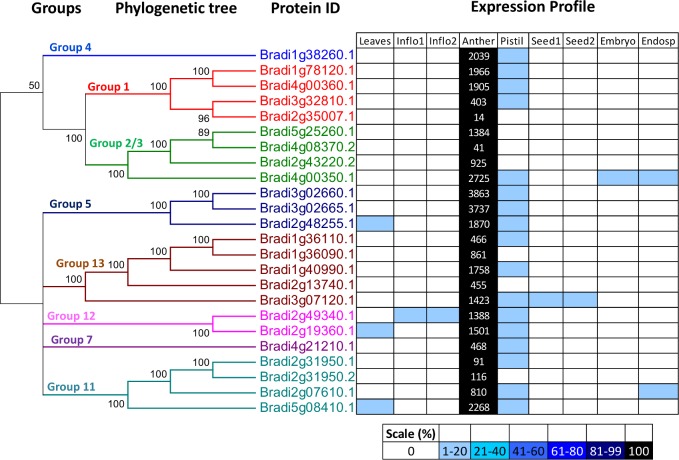
Expression profile and evolutionary pattern of pollen allergens. The heat map shows the relative abundances of putative allergen-encoding genes identified in *B*. *distachyon*. The level of expression for a gene across different samples are represented as percentage of the maximum expression level in colour code from 0% (white) to 100% (black). Heat map was plotted against the phylogenetic tree constructed using *B*. *distachyon* protein sequences. The diagram also shows relative expression values in anther tissue of *B*. *distachyon* based on RNA-Seq data analysis obtained from nine developmental stages. Inflo1 is for pre-emergence flowers stage, Inflo2 is for post-emergence flowers stage, Seed1 is for whole seed at 5days after pollination, Seed2 is for whole seed at 10 days after pollination and Endosp is for endosperm respectively.

A complete gene expression catalogue of *B*. *distachyon* in 9 different tissues is given in S3 Table. Fig 4 illustrates the transcript levels of 24 putative allergen-encodings genes in anther tissues of *B*. *distachyon*. All these 24 genes show strict anther-specificity with some signal in pistil tissues that can be ascribed to RNA contribution from pollen in stigma/styler tissues. The putative isoforms of group 5 (Bradi3g02660.1, Bradi3g02665.1) proteins identified in Brachypodium showed the highest expression values (FPKMs-3863, 3737 respectively).

### Chromosomal distribution and homology modeling

**Fig 5:**
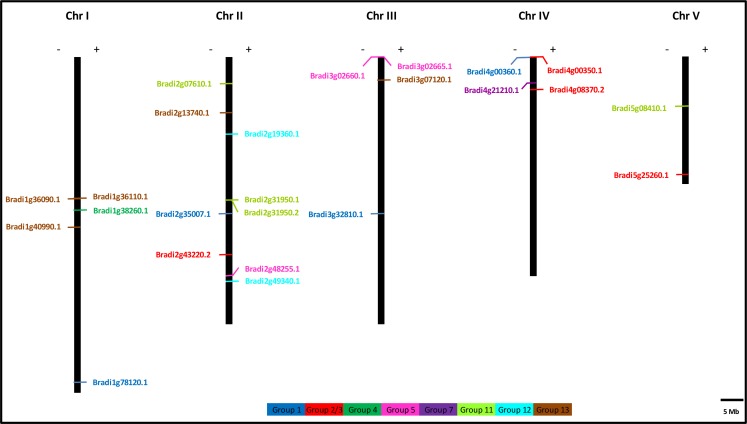
Distribution of 24 putative allergen-encoding genes onto five *B*. *distachyon* chromosomes. The chromosomal position of each gene was mapped according to the *B*. *distachyon* genome. The chromosome number is indicated at the top of each chromosome. The scale is 5 Mb. Genes are marked on the left and right hand side of each chromosome based on their orientation. Genes belonging to different groups of pollen allergens are indicated by the use of different colours as shown at the bottom of the figure.

The precise locations of putative allergen-encoding genes on five chromosomes of *B*. *distachyon* have been illustrated in Fig 5 and their exact positions on *B*. *distachyon* chromosomes are given in S4 Table. Among all, chromosome 2 contained the highest number of allergen-encoding genes (nine) that were unevenly distributed, followed by chromosome 1 and chromosome 3, each containing five genes. Chromosome 4 carried four genes while two genes were located on chromosome 5 (Fig 5). Interestingly, homologs of group 12 were located on chromosome 2 while other groups’ homologs were found to be distributed unevenly among the five chromosomes.

**Fig 6:**
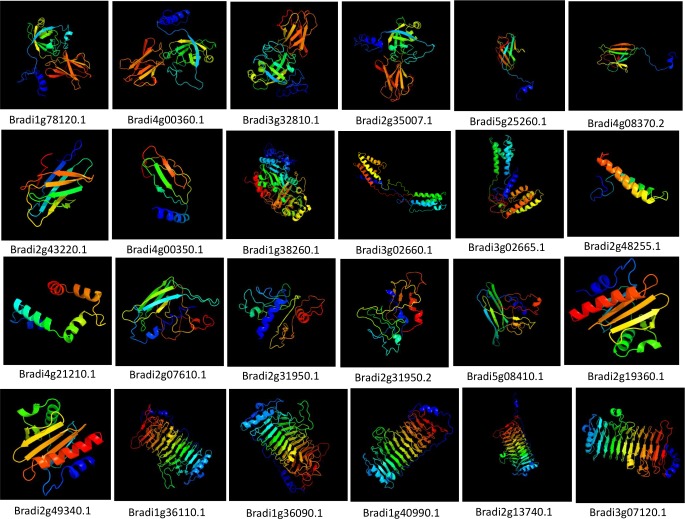
Predicted structures of putative pollen allergen proteins in *B*. *distachyon*. The structure of 24 putative allergen-encoding proteins with >90% confidence level were shown.

To better understand structural characteristics of the allergen proteins, the tertiary structures of *B*. *distachyon* putative pollen allergen proteins were predicted by the Phyre2 server in intensive mode. Fig 6 shows the protein structures that are modelled at >90% confidence. The protein structures clearly differentiate various pollen allergen groups. The secondary structures of putative allergen homologs in Brachypodium predominantly comprised of β-sheets and coils with lesser number of α-helices except for group 5 proteins. As inferred from the predicted tertiary structures (Fig 6), group 1 homologs were shown to form dimers while group 2 homologs contain anti parallel β-strands, possibly forming a β-sandwich. Group 5 proteins were seen as mainly α-helical with each monomer containing a 4-helix bundle. The predicted structures of group 12 proteins (profilins) in Brachypodium were shown to form a main fold comprising of central anti parallel β-sheets and α-helices on both sides. Group 13 proteins were shown to form a central β barrel having several β-strands, with the α-helix situated on each side of the barrel.

### *Cis*-regulatory elements (CREs) of putative allergen-encoding genes of *B*. *distachyon*

**Fig 7:**
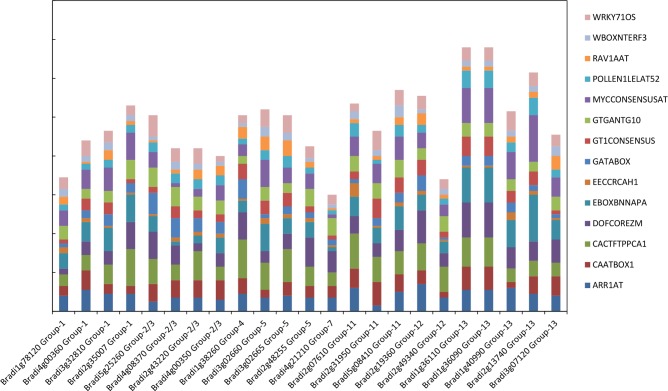
Duplication frequency of 14 most abundant *cis*-regulatory elements (CREs) in putative allergen-encoding genes in *B*. *distachyon*.

A key feature of the 24 genes identified as encoding pollen allergens in the Brachypodium genome is their anther/pollen specific expression. The availability of genomic sequences corresponding to these allergen genes presents an opportunity to identify conserved motifs in their upstream sequences. Evaluation of genomic sequences upstream of the initiation codon of the 24 Brachypodium putative allergen-encoding genes resulted in the identification of 198 types of putative CREs (S5 Table). Fourteen of the CREs were found in all of the 24 genes: ARR1AT (5’- NGATT-3’), CAATBOX1 (5’- CAAT-3’), CACTFTPPCA1 (5’- YACT-3’), DOFCOREZM (5’- AAAG-3’), EBOXBNNAPA (5’- CANNTG-3’), EECCRCAH1 (5’- GANTTNC-3’), GATABOX (5’- GATA-3’), GT1CONSENSUS (5’- GRWAAW-3’), GTGANTG10 (5’- GTGA-3’), MYCCONSENSUSAT (5’- CANNTG-3’), POLLEN1LELAT52 (5’- AGAAA-3’), RAV1AAT (5’- CAACA-3’), WBOXNTERF3 (5’- TGACY-3’) and WRKY71OS (5’- TGAC-3’). The duplication frequency of these CREs in all 24 genes is depicted in Fig 7, and the numbers are given in S5 Table. Among these fourteen CREs, CACTFTPPCA1 is the most abundant CRE, with duplications in the range of 6 to 20 in the 1-kb upstream region of each putative allergen-encoding promoter, followed by DOFCOREZM, MYCCONSENSUSAT and EBOXBNNAPA respectively. In addition to these, some CREs were present uniquely to specific pollen allergen groups such as QELEMENTZMZM13 (5’- AGGTCA-3’) in group 1, SPHCOREZMC1 (5’- TCCATGCAT-3’) in group 2/3, PE2FNTRNR1A (5’- ATTCGCGC-3’) in group 4, REBETALGLHCB21 (5’- CGGATA-3’) and TATAPVTRNALEU (5’- TTTATATA-3’) in group 5, T/GBOXATPIN2 (5’- AACGTG-3’) and QARBNEXTA (5’- AACGTGT-3’) in group 12 respectively. Our results show conservation of putative regulatory sequences in the upstream regions of genes encoding allergens belonging to diverse groups. This provides useful data for the functional characterization of regulatory elements underlying pollen specific expression of allergen-encoding genes.

## Discussion

Grasses have evolved a group or family of allergen encoding genes that appear to be largely restricted to the species of Poaceae. Since genomic data for important allergenic grasses in the family is still unavailable, the genetic information contained in the sequenced genome of model grass *B*. *distachyon* provides a valuable approach to disclose pollen allergen-encoding gene repertoire in grasses and trace their expression profiles and evolutionary relationships. However, some reports on transcriptome- and proteome-based examination of putative pollen allergens in important grasses/cereals are present in the literature [5, 18, 35–39]. Rice was the first member of the angiosperm family Poaceae to be sequenced. An investigation of the rice genome and pollen transcriptome revealed the lack of group 5 allergen genes in this species [37] making it unsuitable as a model for investigating allergen gene repertoire of clinically important pooid grasses such as Ryegrass, Fescue and Timothy grass, etc. Though Brachypodium is not a clinically significant grass because of its short stature and limited pollen production, being a pooid grass, its genome and transcriptome provides a first window for revealing allergen gene repertoire investigating evolutionary and structural biology of grass pollen allergens.

In this study, we have successfully identified 24 putative allergen-encoding proteins in *B*. *distachyon* using genome- and transcriptome-based analysis. To further describe the identified Brachypodium genes, phylogenetic trees and heat maps were constructed which define their evolutionary relationships and depict expression profiles across 9 Brachypodium tissues (Figs 3 and 4). Phylogenetic trees displayed high bootstrap values for internal nodes of different clades and hence, it clearly showed the derivation of statistically reliable pairs of possible homologous proteins sharing similar functions from a common ancestor. The above identified 24 putative allergen-encoding proteins were categorised into eight independent pollen allergen groups depending on the presence of specific domains in their protein sequences (Fig 1). In addition to the identification of putative allergen-encoding genes in *B*. *distachyon*, this study also comprehensively illustrates some important plant gene families for the first time in this model plant like the expansin superfamily, the EF-Hand protein family, Ole e 1 protein family and profilins.

Expansin refers to a family of non-enzymatic proteins found in the plant cell walls with important roles in plant cell growth, developmental processes, and providing resistance to stress [40, 41]. Grass group 1 pollen allergens were recognised as a subgroup of the expansins referred to as β-expansins. Group 1 antigens are classified as major allergens since they cause IgE-mediated sensitization in up to 95% of patients with pollen allergy and exist in multiple isoallergenic forms or isoforms distinguished by their respective isoelectric points and amino acid sequences [1]. The isoform complexity of group 1 allergens and their expression patterns [42] suggest that attempts to silence their expression in a similar manner as have been attempted with the group 5 allergen genes [43] will be challenging. The present survey identified four proteins in *B*. *distachyon* genome belonging to the class of group 1 allergens. Regarding the expression profile, all the four corresponding transcripts showed significantly high expression levels in anther (Fig 4). The expression pattern data will be of particular interest for further experiments in selecting the best candidate for gene silencing approaches and functional validation studies.

Group 5 allergens have been reported to trigger IgE-mediated respiratory allergy symptoms of up to 85% pollen-allergic individuals [1]. Group 5 allergens have also been known to exist in multiple isoforms, and so far have been identified only among the subfamily Pooideae. Group 5 allergen was first discovered as an IgE-binding protein in rye grass pollen cDNA expression library. The cDNA sequence revealed flanking transit peptide sequences typical of plastid-targeted proteins and immunolocalization studies confirmed localisation of allergen protein in amyloplasts of grass pollen [44]. Further immunological cross-reactivity studies showed this allergen group to be restricted to grasses of pooid sub-family establishing why pooid grasses such as ryegrass, timothy grass, Kentucky bluegrass, etc. are clinically most significant grasses. This is in contrast to Panicoid and Chlorodoid grasses with relatively lower allergic potential. Our present study suggests that gene encoding group 5 first appeared during the origin of pooid sub-family. Genomic surveys of the sequenced plant genomes have indeed failed to detect any gene with homology to group 5 protein family. This is in contrast to all other grass pollen allergens whose family members could be observed even in unrelated non-allergenic plant genera. The mechanism of *de novo* origin of group 5 allergen gene in Brachypodium or a related sister species remains a mystery. Earlier it has been reported that in the case of *Drosophila*, *de novo* genes had the most pronounced expression, and many of the *de novo* genes are related to male reproductive processes [45, 46]. Intriguingly grass group 5 allergen genes are also expressed at high levels in male gametophyte-specific manner. It has been proposed that evolutionary split between rice containing clade and Brachypodium containing clade happened around 63 mya [47]. Since rice genome and rice pollen transcriptome does not reveal the presence of group 5 allergen gene but wheat genome shows the presence of group 5 protein, it appears that *de novo* origin of this gene occurred around 50 mya in a common grass ancestor of Brachypodium and Triticiae clades.

Brachypodium pollen allergen-encoding genes were found to be distributed among all five chromosomes following no specific pattern. Some of the genes belonging to the same group of pollen allergen were located on one chromosome suggesting an origin as a result of tandem gene duplication events. For example, homologs of group 12 were solely located on the chromosome 2 (Fig 5). Interestingly, some of the genes exhibited similar gene structures but have different chromosomal locations. For example, both Bradi1g78120.1 and Bradi4g00360.1 (group 1 homologs) had similar exon/intron structures, but they were located on chromosomes 1 and four respectively. This specific distribution suggested that this redistribution may be due to whole grass genome duplication events dating back 96 mya. Similar distribution pattern was also observed in Bradi4g08370.2 and Bradi5g25260.1 (group 2/3 homologs), indicating the unique distribution of pollen allergen genes on Brachypodium chromosomes.

It is a widely accepted that the intron/exon structure of genes is indicative of their evolutionary relationships [48]. Additionally, exon/intron gain/loss is a substantial indication for structural divergence and functional differentiation [49]. All of the transcribed pollen proteins in *B*. *distachyon* were either encoded by loci lacking introns or containing a reduced number of introns but exhibited a high level of expression in anthers. Since introns in some cases may be recruited in post-transcriptional control [50], elimination of introns could potentially release translational regulation, leading to further enhanced expression.

Three-dimensional protein structure models were constructed for 24 putative pollen-allergen proteins using Phyre2 prediction tool (Fig 6). Phye2 uses the alignment of hidden Markov models via HMM-HMM search [51] to significantly improve the accuracy of alignment and detection rate. The intensive mode of Phyre2 uses the multi-template modelling for higher accuracy. Furthermore, it integrates a new *ab initio* folding simulation termed as Poing [52] to model regions of proteins with no noticeable homology to known structures. The protein structure of all the 24 putative pollen allergens was modelled at >90% confidence and the percentage residue varied from 81 to 100. As observed in our study, tertiary structures of group 1 homologs in Brachypodium were shown to form dimers as these proteins consist of two domains. Similar observation was reported for a timothy grass group 1 allergen (Phl p 1) [53]. Likewise, in a previous study, the three-dimensional structure of Phl p 2 was reported to contain an all-β fold with nine anti parallel β-strands that formed a β sandwich [54]. Homology modelling studies for group 5 proteins in Brachypodium showed that these proteins are composed entirely of α-helices. Similar results have been reported earlier for Phl p 5 and Phl p 6 allergens [53, 55]. Hence, all the predicted protein structures were considered highly reliable, and this offers a preliminary basis for understanding the structures of these Brachypodium proteins.

Different *cis*-regulatory elements (CREs) and transcription factors that bind to these CREs have been reported to lie within anther-/pollen- specific promoters and were responsible for anther-/pollen- specific gene expression [56]. Promoter sequences such as GTGANTG10 (5’-GTGA-3’) and POLLEN1LELAT52 (5’- AGAAA-3’) were earlier reported as regulatory elements responsible for activation of anther-/pollen- specific genes [56]. GTGANTG10 is a GTGA motif found in the promoter of tobacco late pollen gene *g10*. The tobacco gene *g10* is preferentially and highly expressed in mature pollen, shows homology to pectate lyases, and is the putative homologue of the tomato gene *lat56* [57]. POLLEN1LELAT52 is a regulatory element responsible for pollen-specific activation of the tomato*lat52* gene. This CRE has been found in the promoter of tomato endo-beta-mannanase gene during late stages of anther development [58]. We have identified identical CREs in the promoter sequences of all the identified Brachypodium allergen homologs. In our study, interestingly, QELEMENTZMZM13, an AGGTCA motif was exclusively found in promoter sequences of putative group 1 allergens. AGGTCA has been reported to be the Quantitative element (Q) responsible for expression enhancing in *Zm13*, a pollen specific gene in maize [59]. Thus, our investigation revealed that the presence of these particular sequences (CREs) in the promoter regions of putative allergen-encoding genes in Brachypodium is responsible for driving high expression in anthers and pollen.

In summary, a total of 24 putative pollen allergen-encoding genes were found to be present in *B*.*distachyon* genome. Comprehensive phylogenetic analysis and sequence alignments with other known pollen allergens classified them into eight independent groups/families of pollen allergens. The variation in the lengths and structures of gene sequences indicated the level of complexity that has evolved within the allergen families. We also described the structure of 24 putative allergen proteins which could expedite the investigation of its molecular functions and facilitate further investigations in to understanding molecular basis of allergenicity. Hence, the present study is the first report on genome-wide identification, characterization and expression profiling of putative pollen allergens in *B*. *distachyon*. It is expected that the data obtained from this study would contribute to a better understanding of the complexity of pollen allergens in related grasses and stimulate further bio-prospecting of grass genomes to search for hypoallergenic forms of allergen proteins.

## Supporting Information

S1 Fig Phylogenetic relationships, gene structures, expression profiles and protein domain architecture of Expansin family in *B*. *distachyon*. The protein sequences were aligned by Clustal X2.0 and unrooted phylogenetic tree was constructed by neighbour-joining method with 100 bootstrap replicates. Branches with less than 50% bootstrap support were collapsed. The tree was divided into four clusters. The members were distinctly coloured to represent respective groups.
(TIF)

S2 Fig Multiple sequence alignment of group 1 homologs in *B*. *distachyon* with group 1 allergen proteins from other grasses. The protein sequences were aligned by Clustal X2.0 and conserved residues were highlighted in different colors.
(DOC)

S3 Fig Phylogenetic relationships, gene structures, expression profiles and protein domain architecture of Group 2/3 homologs in *B*. *distachyon*. The protein sequences were aligned by Clustal X2.0 and unrooted phylogenetic tree was constructed by neighbour-joining method with 100 bootstrap replicates. Branches with less than 50% bootstrap support were collapsed.
(TIF)

S4 Fig Phylogenetic relationships, gene structures, expression profiles and protein domain architecture of Group 2/3 homologs in *B*. *distachyon*. The protein sequences were aligned by Clustal X2.0 and unrooted phylogenetic tree was constructed by neighbour-joining method with 100 bootstrap replicates. Branches with less than 50% bootstrap support were collapsed.
(DOC)

S5 Fig Phylogenetic relationships, gene structures, expression profiles and protein domain architecture of FAD binding and BBE family in *B*. *distachyon*. The protein sequences were aligned by Clustal X2.0 and unrooted phylogenetic tree was constructed by neighbour-joining method with 100 bootstrap replicates. Branches with less than 50% bootstrap support were collapsed. The members were distinctly coloured to represent respective groups.
(TIF)

S6 Fig Multiple sequence alignment of group 4 homologue in *B*. *distachyon* with group 4 allergen proteins from other grasses. The protein sequences were aligned by Clustal X2.0 and conserved residues were highlighted in different colors.
(DOC)

S7 Fig Phylogenetic relationships and protein domain architecture of group 5 proteins in *B*. *distachyon* and other grasses. **Gene structures and expression profiles of group 5 homologs in Brachypodium.** The protein sequences were aligned by Clustal X2.0 and unrooted phylogenetic tree was constructed by neighbour-joining method with 100 bootstrap replicates. Branches with less than 50% bootstrap support were collapsed.
(TIF)

S8 Fig Multiple sequence alignment of group 5 homologs in B. *distachyon* with group 5 allergen proteins from other grasses. The protein sequences were aligned by Clustal X2.0 and conserved residues were highlighted in different colors.
(DOC)

S9 Fig Phylogenetic relationships, gene structures, expression profiles and protein domain architecture of EF-Hand family in *B*. *distachyon*. The protein sequences were aligned by Clustal X2.0 and unrooted phylogenetic tree was constructed by neighbour-joining method with 100 bootstrap replicates. Branches with less than 50% bootstrap support were collapsed.
(TIF)

S10 Fig Multiple sequence alignment of group 7 homologue in *B*. *distachyon* with group 7 allergen proteins from other grasses. The protein sequences were aligned by Clustal X2.0 and conserved residues were highlighted in different colors.
(DOC)

S11 Fig Phylogenetic relationships, gene structures, expression profiles and protein domain architecture of Ole e one family in *B*. *distachyon*. The protein sequences were aligned by Clustal X2.0 and unrooted phylogenetic tree was constructed by neighbour-joining method with 100 bootstrap replicates. Branches with less than 50% bootstrap support were collapsed. Group 11 homologs were represented in blue shade.
(TIF)

S12 Fig Multiple sequence alignment of group 11 homologs in B. *distachyon* with group 11 allergen proteins from other grasses. The protein sequences were aligned by Clustal X2.0 and conserved residues were highlighted in different colors.
(DOC)

S13 Fig Phylogenetic relationships, gene structures, expression profiles and protein domain architecture of Profilin family in *B*. *distachyon*. The protein sequences were aligned by Clustal X2.0 and unrooted phylogenetic tree was constructed by neighbour-joining method with 100 bootstrap replicates. Branches with less than 50% bootstrap support were collapsed. Group 12 homologs were represented in pink shade.
(TIF)

S14 Fig Multiple sequence alignment of group 12 homologs in *B*. *distachyon* with group 12 allergen proteins from other grasses. The protein sequences were aligned by Clustal X2.0 and conserved residues were highlighted in different colors.
(DOC)

S15 Fig Phylogenetic relationships, gene structures, expression profiles and protein domain architecture of Polygalacturonases family in *B*. *distachyon*. The protein sequences were aligned by Clustal X2.0 and unrooted phylogenetic tree was constructed by neighbour-joining method with 100 bootstrap replicates. Branches with less than 50% bootstrap support were collapsed. Group 13 homologs were represented in pink shade.
(TIF)

S16 Fig Multiple sequence alignment of group 13 homologs in *B*. *distachyon* with group 13 allergen proteins from other grasses. The protein sequences were aligned by Clustal X2.0 and conserved residues were highlighted in different colors.
(DOC)

S1 Table Accession numbers and protein sequences of pollen allergens. (XLSX)

S2 Table Percentage of protein sequence coverage and identity of identified Brachypodium proteins with known pollen allergens from other grasses. (XLSX)

S3 Table Gene expression matrix for *Brachypodium distachyon* (mapped against v2.1). (XLSX)

S4 Table Chromosomal location co-ordinates of putative allergen-encoding genes in *B*. *distachyon*. (XLSX)

S5 Table Functional motif numbers of identified *cis*-regulatory elements in putative allergen-encoding genes of *Brachypodium distachyon*. (XLSX)
