# Supplementary figures and images for: Comparative and Evolutionary Analysis of Grass Pollen Allergens Using Brachypodium distachyon as a Model System
Source: PLoS One. 2017 Jan 19;12(1):e0169686. doi: 10.1371/journal.pone.0169686 (PMC5245863; doi:10.1371/journal.pone.0169686)

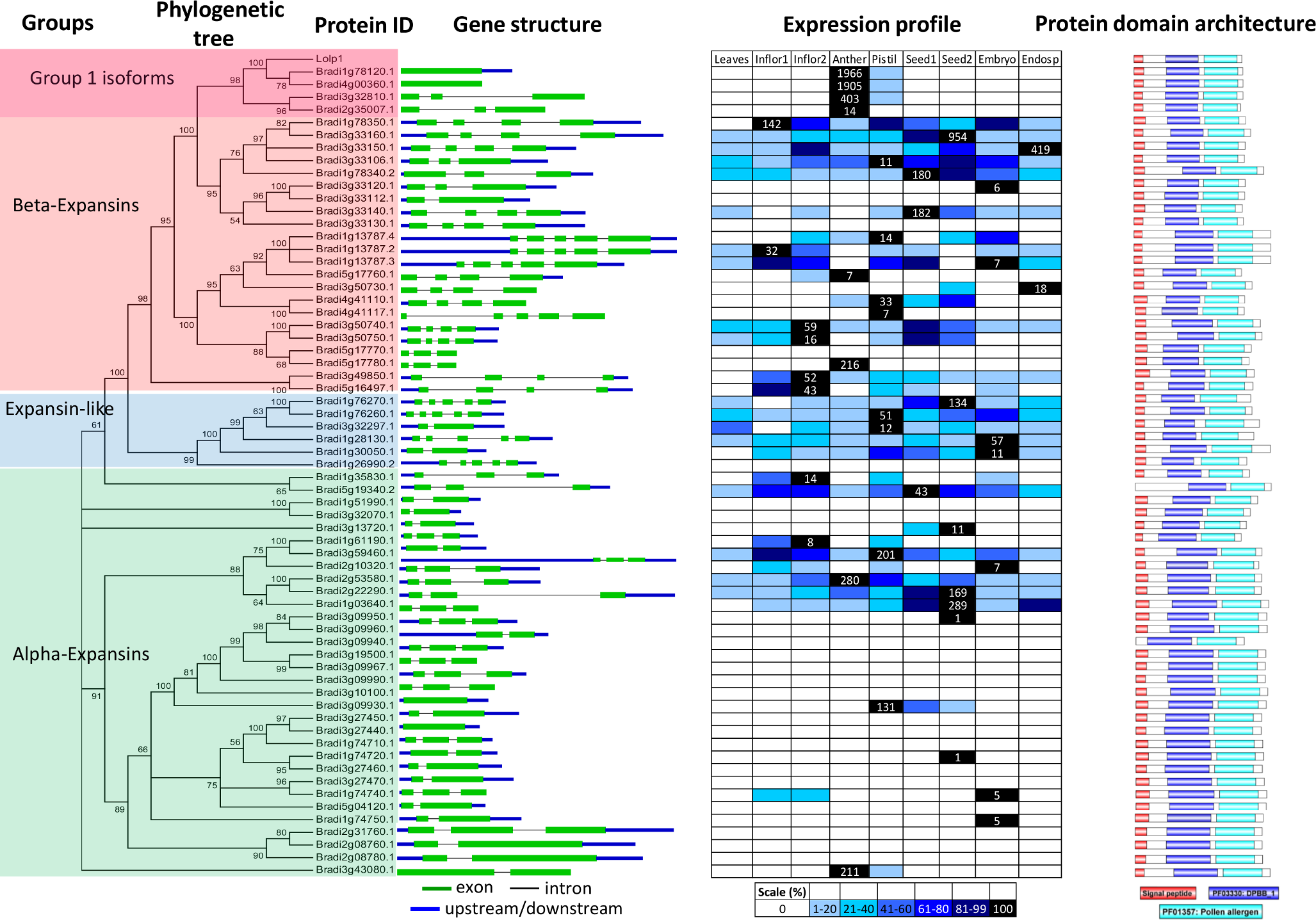

Supplement: S1 Fig — The protein sequences were aligned by Clustal X2.0 and unrooted phylogenetic tree was constructed by neighbour-joining method with 100 bootstrap replicates. Branches with less than 50% bootstrap support were collapsed. The tree was divided into four clusters. The members were distinctly coloured to represent respective groups. (TIF) [file pone.0169686.s001.tif]

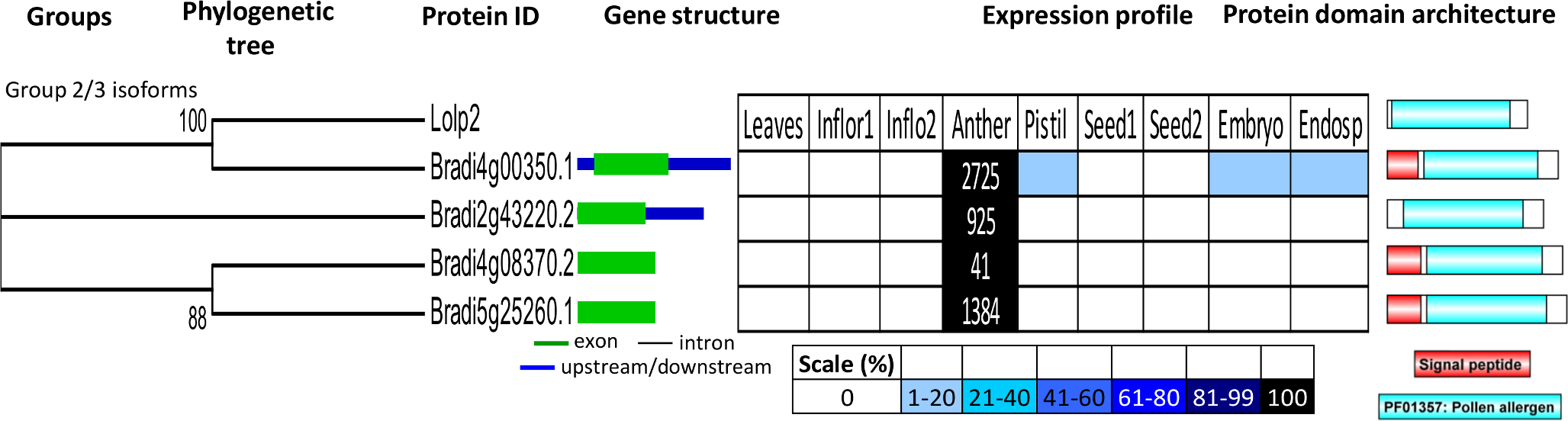

Supplement: S3 Fig — The protein sequences were aligned by Clustal X2.0 and unrooted phylogenetic tree was constructed by neighbour-joining method with 100 bootstrap replicates. Branches with less than 50% bootstrap support were collapsed. (TIF) [file pone.0169686.s003.tif]

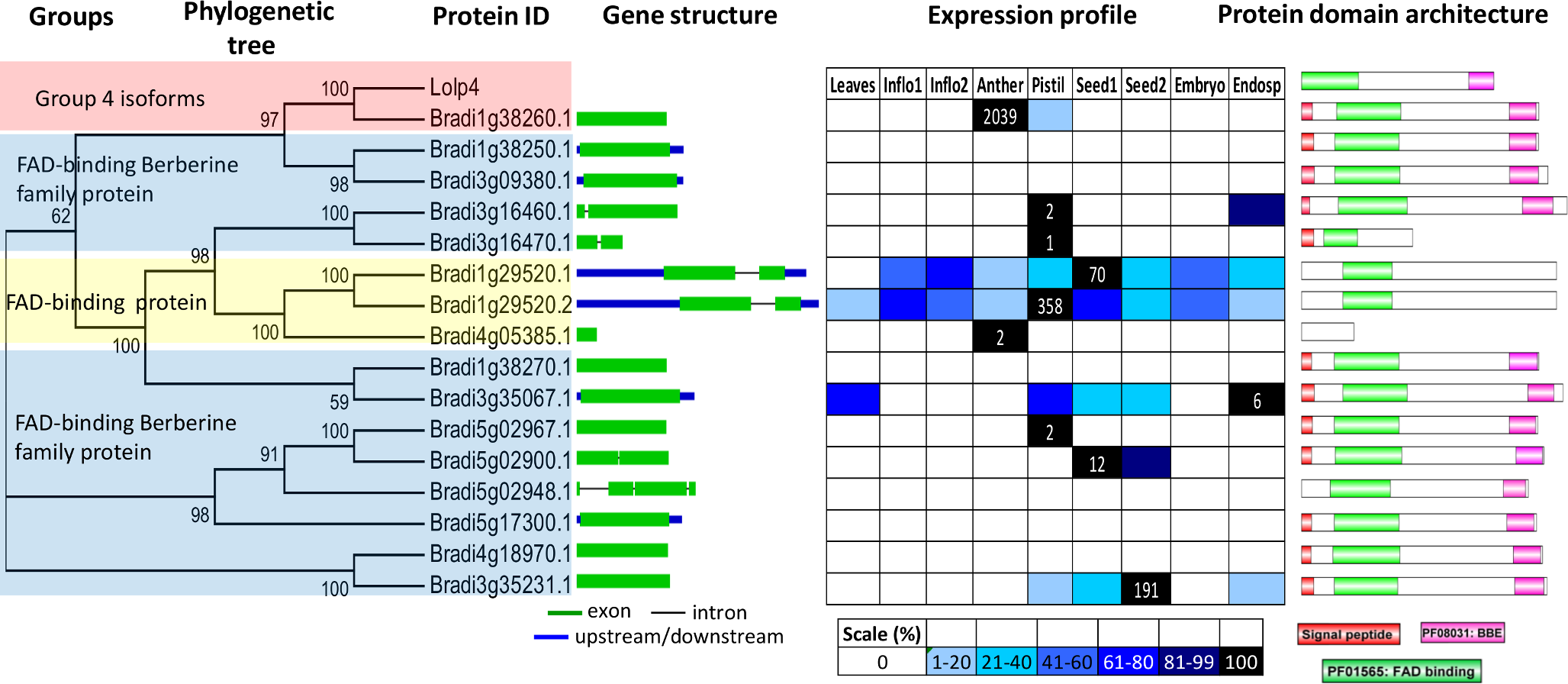

Supplement: S5 Fig — The protein sequences were aligned by Clustal X2.0 and unrooted phylogenetic tree was constructed by neighbour-joining method with 100 bootstrap replicates. Branches with less than 50% bootstrap support were collapsed. The members were distinctly coloured to represent respective groups. (TIF) [file pone.0169686.s005.tif]

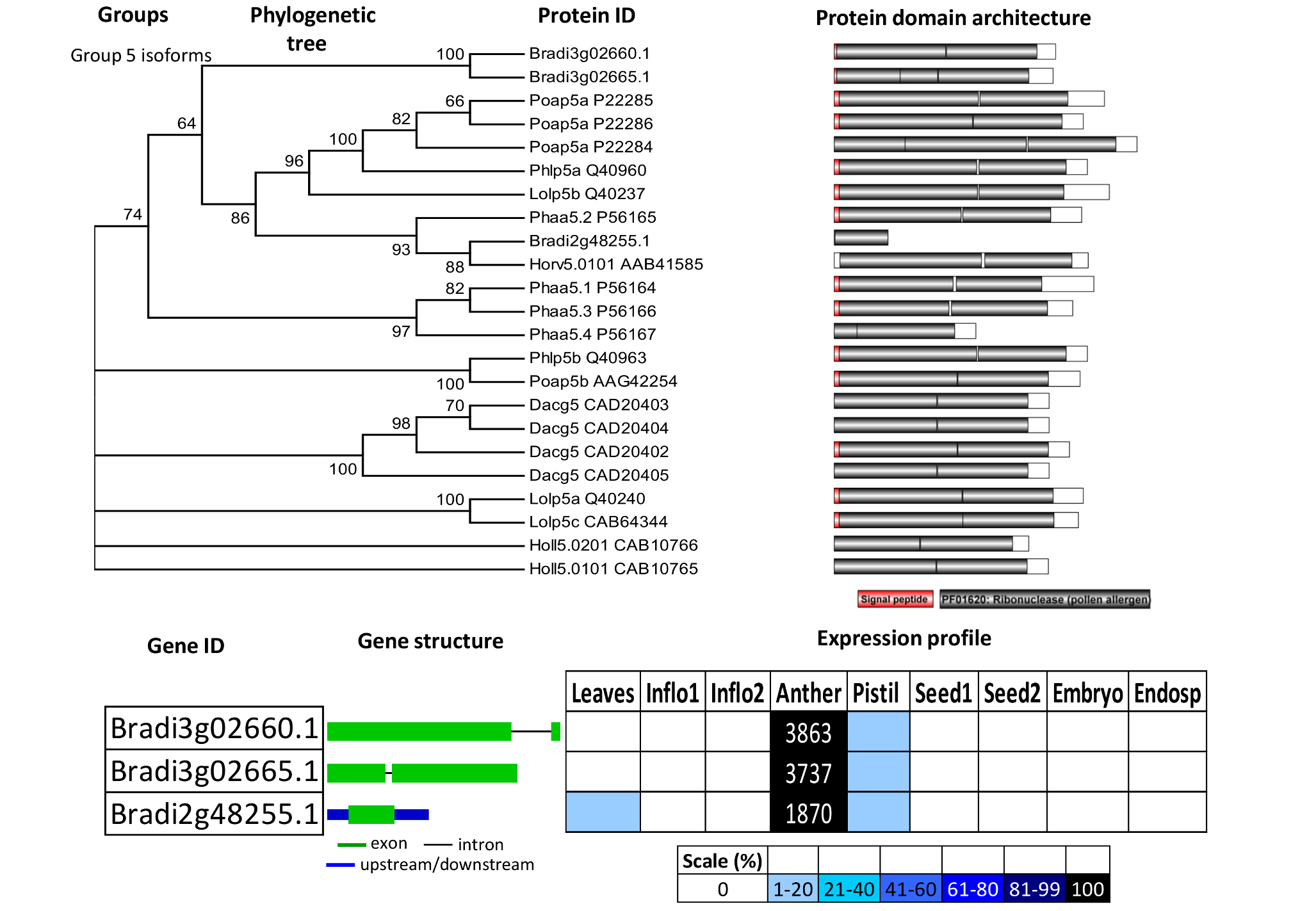

Supplement: S7 Fig — Gene structures and expression profiles of group 5 homologs in Brachypodium. The protein sequences were aligned by Clustal X2.0 and unrooted phylogenetic tree was constructed by neighbour-joining method with 100 bootstrap replicates. Branches with less than 50% bootstrap support were collapsed. (TIF) [file pone.0169686.s007.tif]

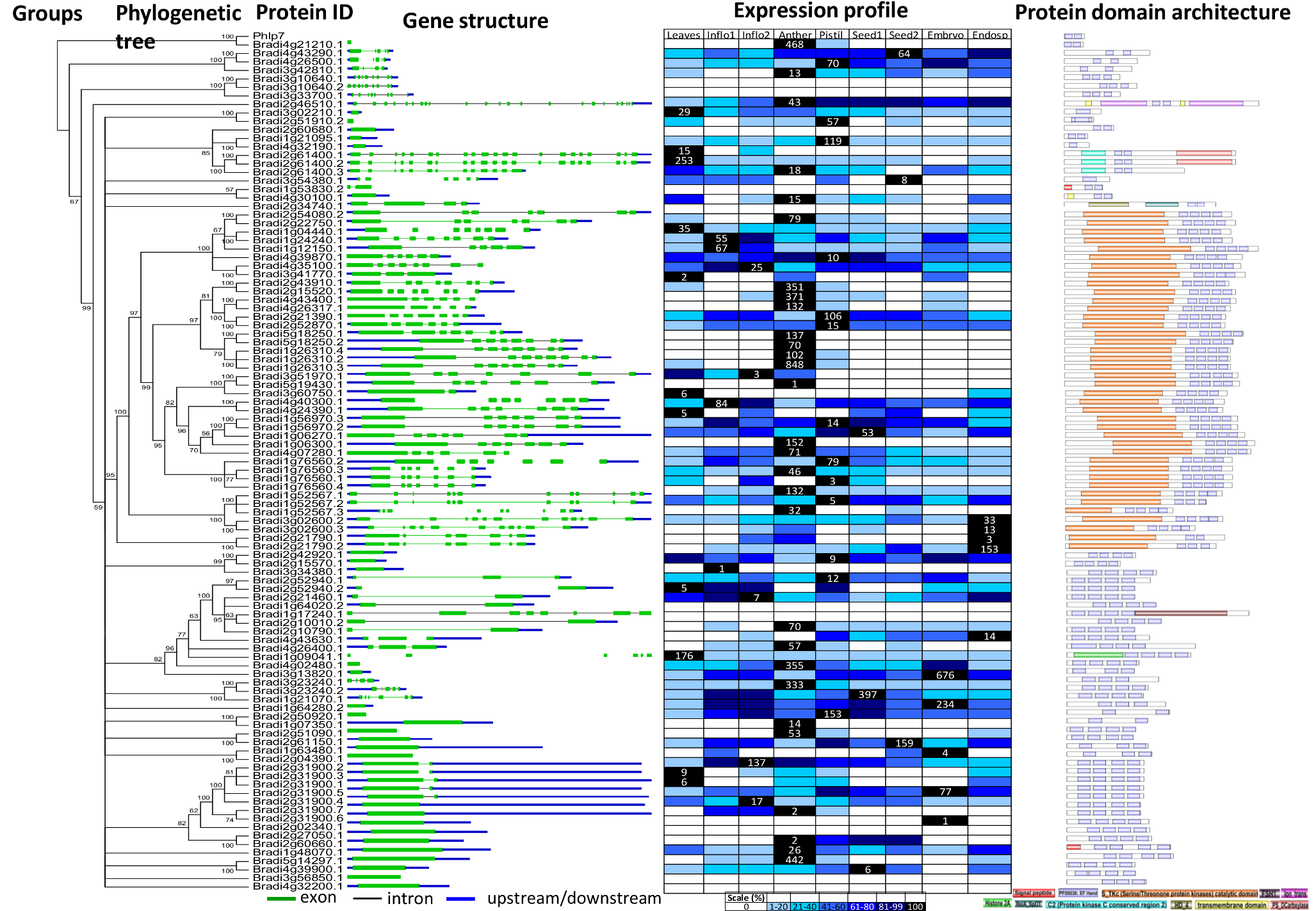

Supplement: S9 Fig — The protein sequences were aligned by Clustal X2.0 and unrooted phylogenetic tree was constructed by neighbour-joining method with 100 bootstrap replicates. Branches with less than 50% bootstrap support were collapsed. (TIF) [file pone.0169686.s009.tif]

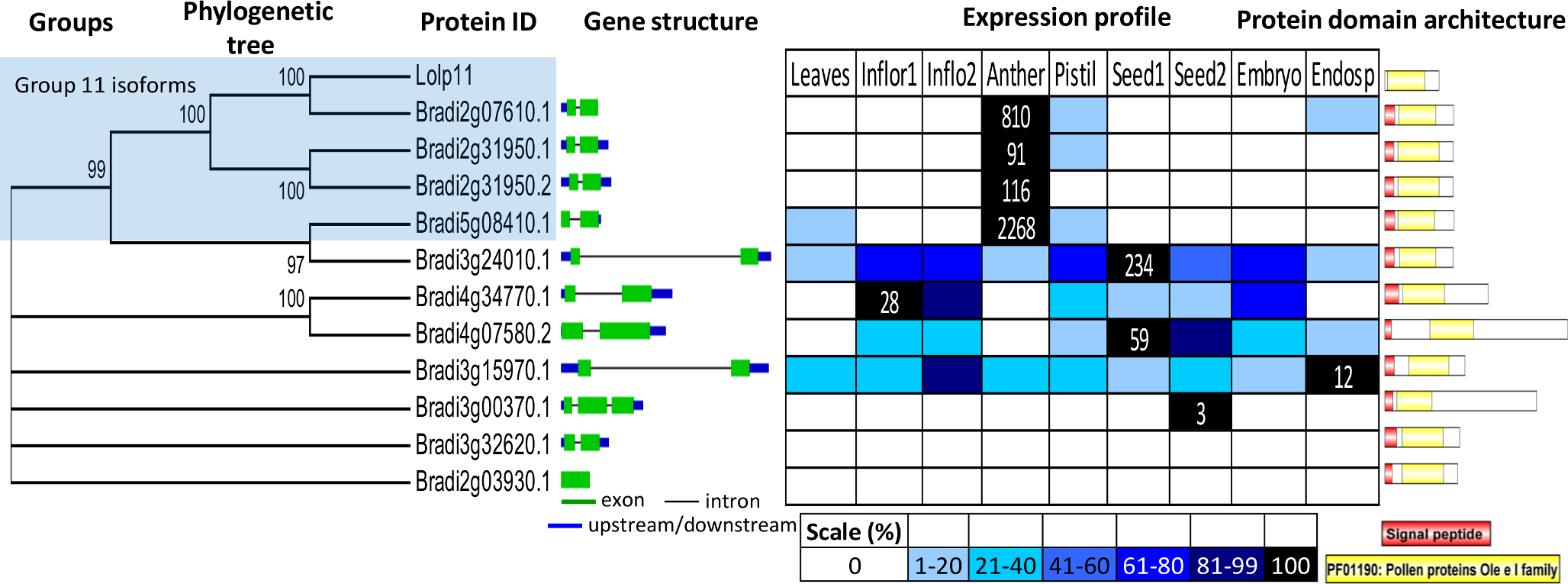

Supplement: S11 Fig — The protein sequences were aligned by Clustal X2.0 and unrooted phylogenetic tree was constructed by neighbour-joining method with 100 bootstrap replicates. Branches with less than 50% bootstrap support were collapsed. Group 11 homologs were represented in blue shade. (TIF) [file pone.0169686.s011.tif]

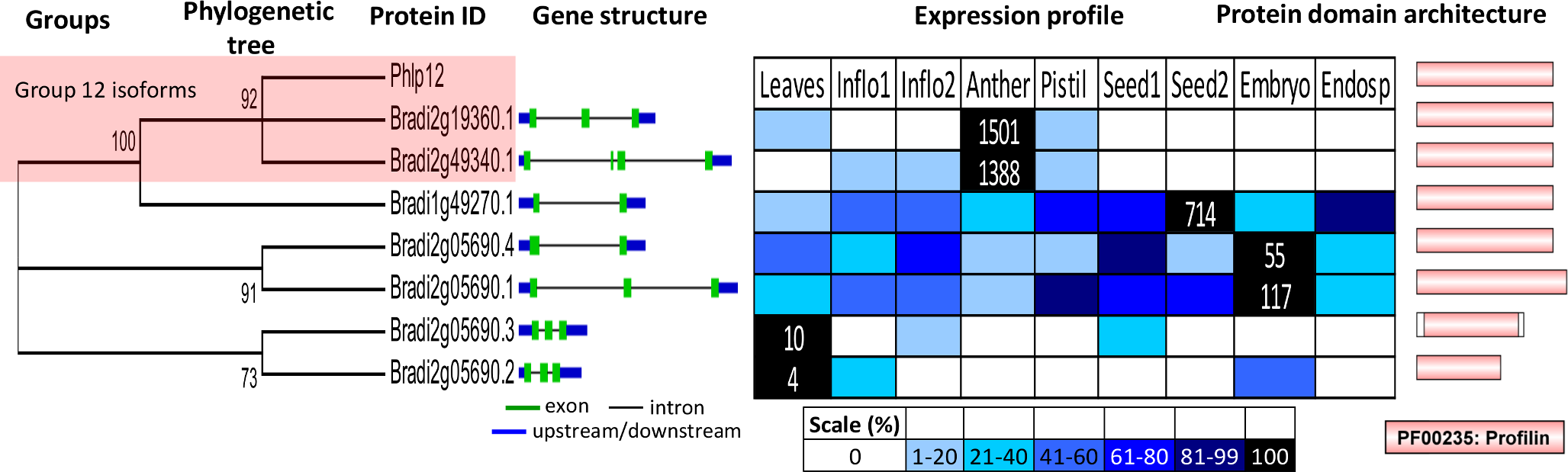

Supplement: S13 Fig — The protein sequences were aligned by Clustal X2.0 and unrooted phylogenetic tree was constructed by neighbour-joining method with 100 bootstrap replicates. Branches with less than 50% bootstrap support were collapsed. Group 12 homologs were represented in pink shade. (TIF) [file pone.0169686.s013.tif]

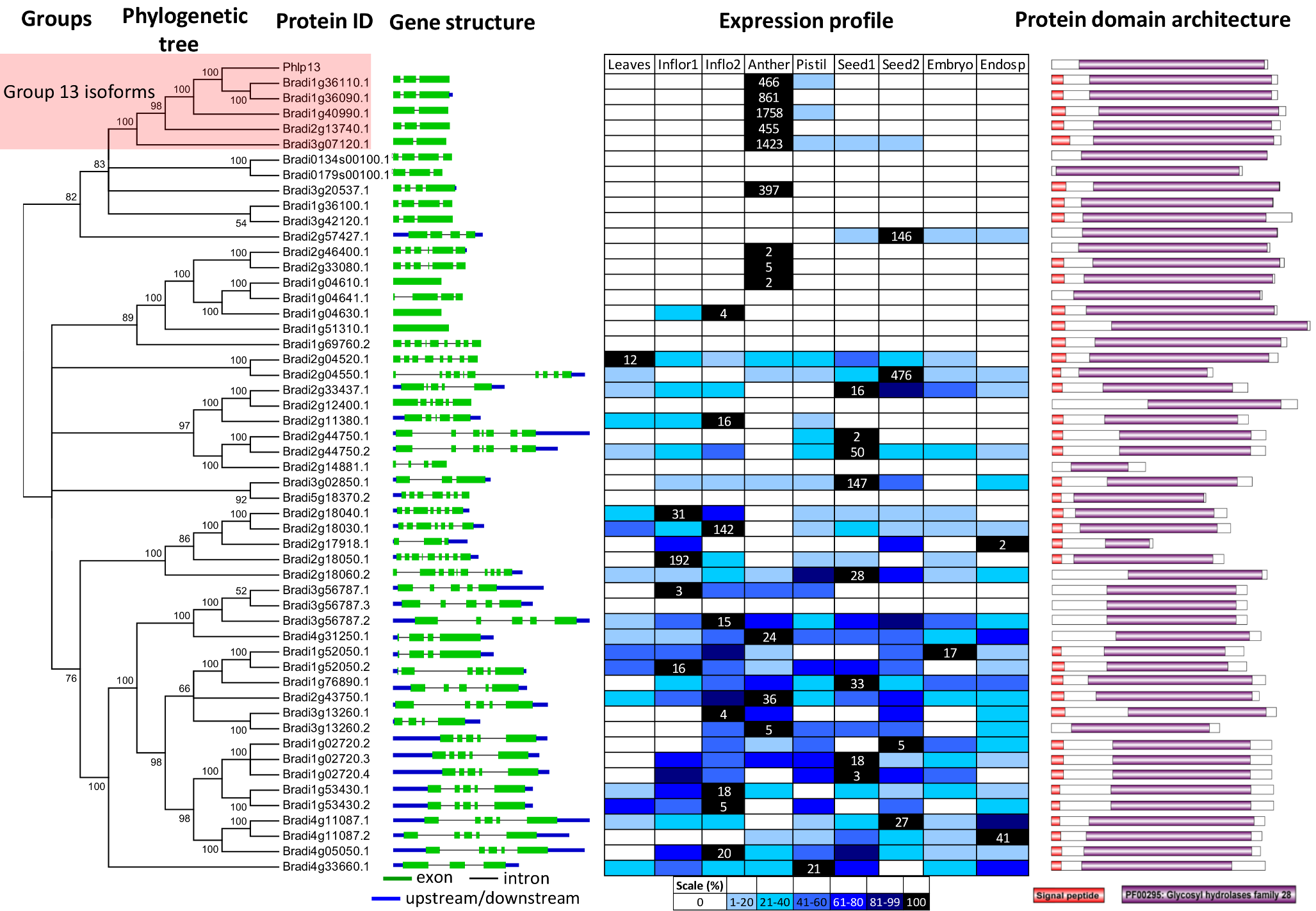

Supplement: S15 Fig — The protein sequences were aligned by Clustal X2.0 and unrooted phylogenetic tree was constructed by neighbour-joining method with 100 bootstrap replicates. Branches with less than 50% bootstrap support were collapsed. Group 13 homologs were represented in pink shade. (TIF) [file pone.0169686.s015.tif]
