# Supplementary material for: Comparative and Evolutionary Analysis of Grass Pollen Allergens Using Brachypodium distachyon as a Model System
Source: PLoS One. 2017 Jan 19;12(1):e0169686. doi: 10.1371/journal.pone.0169686 (PMC5245863; doi:10.1371/journal.pone.0169686)
Supplement: S2 Fig — The protein sequences were aligned by Clustal X2.0 and conserved residues were highlighted in different colors. (DOC) [file pone.0169686.s002.doc]

Lolp1(P14946) IAKVPPGPNITAEYGDKWLDAKSTWYG-KPTGAGPKDNGGACGYKNVDKAPFNGMTGCG

Phaa1(Q41260) IAKVPPGPNITAEYGDKWLDAKSTWYG-KPTGAGPKDNGGACGYKDVDKAPFNGMTGCG

Dacg1(CAD20406) IPKVPPGPNITATYGDKWLDAKSTWYG-KPTGAGPKDNGGACGYKDVDKAPFNGMTGCG

Holl1(P43216) IAKVPPGPNITATYGDEWLDAKSTWYG-KPTGAGPKDNGGACGYKDVDKPPFSGMTGCG

Phlp1(P43213) IPKVPPGPNITATYGDKWLDAKSTWYG-KPTGAGPKDNGGACGYKDVDKPPFSGMTGCG

Poap1(CAA10520) IAKVPPGPNITATYGDKWLDAKSTWYG-KPTGAGPKDNGGACGYKDVDKAPFSGMTGCG

**Bradi1g78120.1** IPKVPPGPNITATYGDKWLDAKSTWYG-KPTGAGPKDNGGACGYKDVDKEPFSGMTSCG

**Bradi4g00360.1** IPKVPPGPNITATYNGKWLDAKSTWYG-RPEGAGPKDNGGACGYKDVDKPPFNGMTSCG

Orys1(AAA86533) GHPKVPPGPNITTSYGDKWLEARPPGMV-RPRVLAPKDNGGACGYKDVDKAPFLGMNSCG

Cynd1(O04701) AIGDKPGPNITATYGSKWLEARATFYGSNPRGAAPDDHGGACGYKDVDKPPFDGMTACG

Zeam1(P58738) GPPKVPPGPNITTNYNGKWLTARATWYG-QPNGAGAPDNGGACGIKNVNLPPYSGMTACG

**Bradi3g32810.1** IPKVPPGPNVTANYNGQWLNAMATWYG-KPTGAGPKDNGGACGIKDVNLPPYSAMTACG

**Bradi2g35007.1** FVVVPPGPNITTSYDGHWLHAKATWYG-SPTGAGPPDNGGSCGIKETNLPPYNSLTSCG

Lolp1(P14946) NTPIFKDGRGCGSCFEIKCTKP-ESCSGEAVTVTITDDNEEPIAPYHFDLSGHAFGSMAK

Phaa1(Q41260) NTPIFKDGRGCGSCFELKCSKP-ESCSGEPITVHITDDNEEPIAPYHFDLSGHAFGSMAK

Dacg1(CAD20406) NTPIFKDGRGCGSCFEIKCTKP-ESCSGEAVTVHITDDNEEPIAPYHFDLSGHAFGSMAK

Holl1(P43216) NTPIFKDGRGCGSCFEIKCTKP-ESCSGEPVTVHITDDNEEPIAPYHFDLSGHAFGSMAK

Phlp1(P43213) NTPIFKSGRGCGSCFEIKCTKP-EACSGEPVVVHITDDNEEPIAPYHFDLSGHAFGAMAK

Poap1(CAA10520) NTPIFKSGRGCGSCFEIKCTKP-ESCSGEPVLVHITDDNEEPIAAYHFDLSGKAFGAMAK

**Bradi1g78120.1** NTPIFRDGRGCGSCFEIKCTKP-DACSGDPVLVHITDDNEEPIAAYHFDLSGHAFGSMAK

**Bradi4g00360.1** NTPIFRDGRGCGSCFEIKCDKPAEFCSGQPVLVHITDDNEEPIAAYHFDLSGKAFGSMAK

Orys1(AAA86533) NDPIFKDGKGCGSCFEIKCSKP-EACSDKPALIHVTDMNDEPIAAYHFDLSG---LAMAK

Cynd1(O04701) NEPIFKDGLGCRACYEIKCKEP-VECSGEPVLVKITDKNYEHIAAYHFDLSGKAFGAMAK

Zeam1(P58738) NVPIFKDGKGCGSCYEVRCKE-KPECSGNPVTVYITDMNYEPIAPYHFDLSGKAFGSLAK

**Bradi3g32810.1** NVPIFKDGRGCGSCYEVKCNVPAKLCSNKPITVFITDMNYEPIAPYHLDLSGTAFGLMAQ

**Bradi2g35007.1** NLPIFKDGKGCGSCFKIKCGN—-KVCSKKPVVVFITDMNYYQFAPYHFDLSGTAFGALAM

Lolp1(P14946) KGEEQNVRSAGELELQFRRVKCKYPDDTKPTFHVEKASNPNYLAILVKYVDGDGDVVAVD

Phaa1(Q41260) KGEEENVRGAGELELQFRRVKCKYPDGTKPTFHVEKGSNPNYLALLVKYVDGDGDVVAVD

Dacg1(CAD2040) KGEEQKLRSAGELELQFRRVKCKYPEGTKVTFHVEKGSNPNYLALLVKYVDGDGDVVAVD

Holl1(P43216) KGEEQKLRSAGELELKFRRVKCKYPDGTKPTFHVEKGSNPNYLALLVKYIDGDGDVVAVD

Phlp1(P43213) KGDEQKLRSAGELELQFRRVKCKYPEGTKVTFHVEKGSNPNYLALLVKYVNGDGDVVAVD

Poap1(CAA1052) KGEEQKLRSAGELELKFRRVKCEYPEGTKVTFHVEKGSNPNYLALLVKYVTGDGDVVAVD

**Bradi1g78120.1** KGKEQDLRSAGEVEIQFRRVKCKYPEGTKVTFHVEKGSSPNYLAILVKYVGGDGDVVAVD

**Bradi4g00360.1** KGQEQKLRGCGEVEIQFRRVKCYYPLGTKVTYHVEKGSNPNYLALLVKFVGGDGDVVAVE

Orys1(AAA86533) DGKDEELRKAGIIDTQFRRVKCKYPADTKITFHIEKASNPNYLALLVKYVAGDGDVVEVE

Cynd1(O04701) KGQEDKLRKAGELTLQFRRVKCKYPSGTKITFHIEKGSNDHYLALLVKYAAGDGNIVAVD

Zeam1(P58738) PGLNDKIRHCGIMDVEFRRVRCKYPAGQKIVFHIEKGCNPNYLAVLVKYVADDGDIVLME

**Bradi3g32810.1** PGKEQLLRNCGELQLQFRRVRCKLPPGTKITFHIEKGSNPNYLAVLVKFASDDGDIVQMD

**Bradi2g35007.1** PGRESELRRYGIIDLRFRRVRCKLAPKMKIAFHVEEGSNPEYLAVLVKFVAGDGDIVQMD

Lolp1(P14946) IKEKGKDKWIELKESWGAVWRIDT--PDKLTGPFTVRYTTEGGTKSEFEDVIPEGWKADT

Phaa1(Q41260) IKEKGKDKWIELKESWGAIWRIDT--PDKLTGPFTVRYTTEGGTKAEFEDVIPEGWKADT

Dacg1(CAD2040) IKEKGKDKWIALKESWGAIWRVDT--PDKLTGPFTVRYTTEGGTKSEVEDVIPEGWKADT

Holl1(P43216) IKEKGKDKWIELKESWGAVWRVDT--PDKLTGPFTVRYTTEGGTKGEAEDVIPEGWKADT

Phlp1(P43213) IKEKGKDKWIELKESWGAIWRIDT--PDKLTGPFTVRYTTEGGTKTEAEDVIPEGWKADT

Poap1(CAA1052) IKEKGKDKWIELKESWGSIWRVDT--PDKLTGPFTVRYTTEGGTKGEAEDVIPEGWKADT

**Bradi1g78120.1** VKEKGKDEWVALKESWGAVWRLDT--AKPLKGPLTVRYTTDGGTKGESEDVIPEDWKPDT

**Bradi4g00360.1** VQEKGKYNWIPLKESWGAVWRIDT--AKPLKGPLSVRYTTDGGTKAVSPDVIPEKWKPDT

Orys1(AAA86533) IKEKGSEEWKALKESWGAIWRIDT--PKPLKGPFSVRVTTEGARRSSAEDAIPDPGRRQR

Cynd1(O04701) IKPRDSDEFIPMKSSWGAIWRIDP--KKPLKGPFSIRLTSEGGAHLVQDDVIPANWKPDT

Zeam1(P58738) IQDKLSAEWKPMKLSWGAIWRMDT--AKALKGPFSIRLTSESGKKVIAKDVIPANWRPDA

**Bradi3g32810.1** LQDKISPEWKPMKESWGAVWRMDS—-IKPLKGPYSIRLTSESGKKLIAKDIIPLNWKPDT

**Bradi2g35007.1** LKQEGWPEWKPMRESWGAIWRMDVD--HPLQGPFSIRLTSESGKVLVAPHVIPADWKPKT

Lolp1(P14946) SYSAK-----

Phaa1(Q41260) HDASK-----

Dacg1(CAD2040) SYEAK-----

Holl1(P43216) AYEAK-----

Phlp1(P43213) SYESK-----

Poap1(CAA1052) AYASK-----

**Bradi1g78120.1** MYESK-----

**Bradi4g00360.1** MYVAKY----

Orys1(AAA86533) VQVNVQAK--

Cynd1(O04701) VYTSKLQFGA

Zeam1(P58738) VYTSNVQFY-

**Bradi3g32810.1** FLKSNIQF—

**Bradi2g35007.1** --------

Yellow: hydroxyproline residues at positions 5 and 8 Light Gray: Conserved residue

Pink: N-glycosylation site Dark Gray: Conserved substitutions

Green: 7 conserved cysteine residues Red: IgE-binding residues
