# Supplementary material for: Comparative and Evolutionary Analysis of Grass Pollen Allergens Using Brachypodium distachyon as a Model System
Source: PLoS One. 2017 Jan 19;12(1):e0169686. doi: 10.1371/journal.pone.0169686 (PMC5245863; doi:10.1371/journal.pone.0169686)
Supplement: S4 Fig — The protein sequences were aligned by Clustal X2.0 and unrooted phylogenetic tree was constructed by neighbour-joining method with 100 bootstrap replicates. Branches with less than 50% bootstrap support were collapsed. (DOC) [file pone.0169686.s004.doc]

Poap2(CAA10348) --------VPKVTFTVEKGSNEK--HLAVLVKYEGDTMAEVELREHGSDEWVAMTKGEGG

Holl2(CAA10347) --------VPKVTFTVEKGSNEK--HLAVLVKYEGDTMAEVELREHGSDEWVAMTKGEGG

Phlp2(P43214) --------VPKVTFTVEKGSNEK--HLAVLVKYEGDTMAEVELREHGSDEWVAMTKGEGG

Dacg2(CAA10345) --------VPKVTFTVEKGSNEK--HLAVLVKYEGDTMAEVELREHGSDEWVAMTKGEGG

Tria2(CAA10349) --------VPKVTFTVEKGSNEK--HLAVLVKYEGDTMAEVELREHGSDEWVAMTKGEGG

Cynd2(CAA10346) --------VPKVTFTVEKGSNEK--HLAVLVKYEGDTMAEVELREHGSDEWVAMTKGEGG

**Bradi4g00350.1** ---------VKPTFTVQPGSTTK--KLGVKVNKPGHSVAEVELRQHGSETWLTMKKTGPD

**Bradi4g08370.2** --------APKVTFTVEKGSDPK---KIVKYDKEGDGMAEVELKQKGSNEWLAMSKCKDT

**Bradi5g25260.1** --------APKVTFTVEKGSDPKKIVLQVKYDKEGDSMAEVELKQKGSNEWLAMSKCKDT

**Bradi2g43220.2** MLFAGVWCAPKVTFTVEKGSDPKKIVLDVKYNKEGDSMAEVELKQKGSNEWLAMSKCKDT

Dacg3(P93124) ---------VKVTFKVEKGSDPKKLVLDIKYTRPGDTLAEVELRQHGSEEWEPLTKKGN-

Lolp3(P14948) ---------TKVDLTVEKGSDAKTLVLNIKYTRPGDTLAEVELRQHGSEEWEPMTKKGN-

Lolp2 (P14947) --------AAPVEFTVEKGSDEKNLALSIKYNKEGDSMAEVELKEHGSNEWLALKKNGDG

Poap2(CAA10348) -VWTFDSEEPLQGPFNFRFLTEKGMKNVFDDVVPEKYTIGATYAPEE-

Holl2(CAA10347) -VWTFDSEEPLQGPFNFRFLTEKGMKNVFDDVVPEKYTIGATYAPEE-

Phlp2(P43214) -VWTFDSEEPLQGPFNFRFLTEKGMKNVFDDVVPEKYTIGATYAPEE-

Dacg2(CAA10345) -VWTFDSEEPLQGPFNFRFLTEKGMKNVFDDVVPEKYTIGATYAPEE-

Tria2(CAA10349) -VWTFDSEEPLQGPFNFRFLTEKGMKNVFDDVVPEKYTIGATYAPEE-

Cynd2(CAA10346) -VWTFDSEEPLKGPFNFRFLTEKGMKNVFDDVVPEKYTIGATYAPEE-

**Bradi4g00350.1** -TFTVQSPTPLKGPYNFRIVTEKGLRGVFDDVVPETFKCGTTYVPDEY

**Bradi4g08370.2** GAWKYESPEPPKCPLNIRFQSEKGMRNVFDDVIPENYKIGSSYAPQEY

**Bradi5g25260.1** GAWKYESPEPLKCPLNIRFQSEKGMRNVFDDVIPENYKIGSTYSPQEY

**Bradi2g43220.2**  GVWKYESPEALKCPLNIRFQSEKGMRNVFDDVIPENYKVGSTYAPQEY

Dacg3(P93124) -LWEVKSSKPLTGPFNFRFMSKGGMRNVFDEVIPTAFKIGTTYTPEE-

Lolp3(P14948) -LWEVKSAKPLTGPMNFRFLSKGGMKNVFDEVIPTAFTVGKTYTPEYN

Lolp2 (P14947) -VWEIKSDKPLKGPFNFRFVSEKGMRNVFDDVVPADFKVGTTYKPE--

Red: fully conserved epitope residues

Turquoise: conservatively substituted epitope residues

Light gray: conserved residues

Dark gray: conservative substitutions
