# Supplementary material for: Comparative and Evolutionary Analysis of Grass Pollen Allergens Using Brachypodium distachyon as a Model System
Source: PLoS One. 2017 Jan 19;12(1):e0169686. doi: 10.1371/journal.pone.0169686 (PMC5245863; doi:10.1371/journal.pone.0169686)
Supplement: S6 Fig — The protein sequences were aligned by Clustal X2.0 and conserved residues were highlighted in different colors. (DOC) [file pone.0169686.s006.doc]

Lolp4(Q5T1W3) ------------------------------------------------------------

Phlp4.0201(CAD54671) SSCQVAFSYFPPPAAKEDFLGCLVKEIPPRLLYAKSSPAYPSVLGQTIRNSRWSSPDNVK

Phlp4.0101(CAD54670) SSCEVALSYYPTPLAKEDFLRCLVKEIPPRLLYAKSSPAYPSVLGQTIRNSRWSSPDNVK

Tria4.0101(CAH92632) --------TYAPVPAKEDFLGCLMKEIPARLLYAKSSPDFPTVLAQTIRNSRWLSPQNVK

Tria4.0201(CAH92633) --------TYAPVPAKEDFLGCLMKEIPARLLYAKSSPDYPTVLAQTIRNSRWSTQQNVK

Secc4.0101(CAH92627) -------AAYAPVPAKADFLGCLMKEIPARLLYAKSSPDYPTVLAQTIRNSRWSSPQNVK

Secc4.0201(CAH92630) ----AVSYAAAPVPAKEDFFGCLVKEIPARLLYAKSSPAFPTVLAQTIRNSRWSSPQSVK

Bradi1g38260.1 --------YPPVLAPKEDFLACLVKEIPPRLLYAKSSPAYPTVLSATIRNSRWSSPQNVK

Lolp4(Q5T1W3) ---------------AVVCGRRYDVRIRVRSGGHDYEGLSYRSL-QPENFAVVDLNQMRA

Phlp4.0201(CAD54671) PLYIITPTNVSHIQSAVVCGRRHSVRIRVRSGGHDYEGLSYRSL-QPETFAVVDLNKMRA

Phlp4.0101(CAD54670) PIYIVTPTNASHIQSAVVCGRRHGVRIRVRSGGHDYEGLSYRSL-QPEEFAVVDLSKMRA

Tria4.0101(CAH92632) PLYIITPTNASHIQSAVVCGRRHSVRLRVRSGGHDYEGLSYRSE-KPETFAVVDLNKMRA

Tria4.0201(CAH92633) PLYIITPTNASHIQSAVVCGRRHGVRLRVRSGGHDYEGLSYRSE-KPETFAVVDLNKMRA

Secc4.0101(CAH92627) PIYIITPTNASHIQSAVVCGRRHGIRLRVRSGGHDYEGLSYRSE-KPETFAVVDLNKMRA

Secc4.0201(CAH92630) PLYIITPTNASHIQSAVVCGRRHGVRIRVRSGGHDYEGLSYRSE-RPEAFAVVDLNKMRA

Bradi1g38260.1 PLYIVTPTNVSHIQSAVVCGRRHGVRIRVRSGGHDYEGLSYRSE-RAESFAVVDLNMMRA

Lolp4 (Q5T1W3) VLVDGKARTAWVDSGAQLGELYYAISKYS-RTLAFPAGVCPTIGVGGNLAGGGFGMLLRK

Phlp4.0201(CAD54671) VWVDGKARTAWVDSGAQLGELYYAIYKAS-PTLAFPAGVCPTIGVGGNFAGGGFGMLLRK

Phlp4.0101(CAD54670) VWVDGKARTAWVDSGAQLGELYYAIHKAS-PVLAFPAGVCPTIGVGGNFAGGGFGMLLRK

Tria4.0101(CAH92632) VLIDGYARTAWVESGAQLGELYYAIAKNS-PVLAFPAGVCPTIGVGGNFAGGGFGMLLRK

Tria4.0201(CAH92633) VVVDGYARTAWVESGAQLGELYYAIAKNS-PVLAFPAGVCPSIGVGGNFAGGGFGMLLRK

Secc4.0101(CAH92627) VSVDGYARTAWVESGAQLGELYYAIAKNS-PVLAFPAGVCPSIGVGGNFAGGGFGMLLRK

Secc4.0201(CAH92630) VVVDGKARTAWVDSGAQLGELYYAIAKNS-PVLAFPAGVCPTIGVGGNFAGGGFGMLLRK

Bradi1g38260.1 VSVDANARTAWVESGAQIGELYYAISKAS-PSLAFPAGVCPSIGVGGHFSGGGFGMLLRK

Lolp4(Q5T1W3) YGIAAENVIDVKLVDANGKLHDKKSMGDDHFWAVRGGGG-ESFGIVVSWQVKLLPVPPTV

Phlp4.0201(CAD54671) YGIAAENVIDVKLVDANGKLHDKKSMGDDHFWAVRGGGG-ESFGIVVAWQVKLLPVPPTV

Phlp4.0101(CAD54670) YGIAAENVIDVKLVDANGTLHDKKSMGDDHFWAVRGGGG-ESFGIVVAWKVRLLPVPPTV

Tria4.0101(CAH92632) YGIAAENVIDVKVVDPNGKLLDKSSMSPDHFWAVRGGGG-ESFGIVVSWQVKLLPVPPTV

Tria4.0201(CAH92633) YGIAAENVIDVKVVDPDGKLLDKSSMSADHFWAVRGGGG-ESFGIVVSWQVKLMPVPPTV

Secc4.0101(CAH92627) YGIAAENVIDVKVVDPNGKLLDKSSMSADHFWAVRGGGG-ESFGIVVSWQVKLLPVPPTV

Secc4.0201(CAH92630) YGIAAENVIDVKVVDANGTLLDKSSMSADHFWAVRGGGG-ESFGIVVSWQVKLLPVPPTV

Bradi1g38260.1 FGIAAENVLDAKLVDANGKLHDRKSMGEDHFWAIRGGGG-ESFGIVVGWEVKLLPVPPVV

Lolp4(Q5T1W3) TIFKIPKSVSEGAVDIINKWQLVAPQLPADLMIRIIAMGPKATFEAMYLGTCKTLTPMMQ

Phlp4.0201(CAD54671) TIFKISKTVSEGAVDIINKWQVVAPQLPADLMIRIIAQGPKATFEAMYLGTCKTLTPLMS

Phlp4.0101(CAD54670) TVFKIPKKASEGAVDIINRWQVVAPQLPDDLMIRVIAQGPTATFEAMYLGTCQTLTPMMS

Tria4.0101(CAH92632) TVFKIPKTVQEGAVDLVNKWQLVGPALPGDLMIRVIAAGNTATFEGMYLGTCQTLTPLMS

Tria4.0201(CAH92633) TVFKIPKTVQEGAVDLVNKWQLVGPALPGDLMIRVIAAGNTATFEALYLGTCKTLTPLMS

Secc4.0101(CAH92627) TVLKIPKTVQEGAIDLVNKWQLVGPALPGDLMIRIILAGNSATFEAMYLGTCSTLTPLMS

Secc4.0201(CAH92630) TVFKIPKTVQEGAVELINKWQLVAPALPDDLMIRIIAFGGTAKFEAMYLGTCKALTPLMS

Bradi1g38260.1 TVFKVSKTLKDGAIDIVNKWQTVAPALPGDLMIRILAMAQQATFEGMYLGTCNNLLPLIT

Lolp4(Q5T1W3) SKFPELGMNASHCNEMSWIESIPFVHLGHRDSLEGDLLNRNNTFKPFAEYKSDYVYEPFP

Phlp4.0201(CAD54671) SKFPELGMNPSHCNEMSWIQSIPFVHLGHRDALEDDLLNRNNSFKPFAEYKSDYVYQPFP

Phlp4.0101(CAD54670) SKFPELGMNASHCNEMSWIQSIPFVHLGHRDNIEDDLLNRNNTFKPFAEYKSDYVYEPFP

Tria4.0101(CAH92632) SQFPELGMNPYHCNEMPWIKSIPFIHLG-KEASLVDLLNRNNTFKPFAEYKSDYVYQPFP

Tria4.0201(CAH92633) SQFPELGMNPYHCNEMPWIKSVPFIHLG-KQAGLDDLLNRNNTFKPFAEYKSDYVYQPFP

Secc4.0101(CAH92627) SKFPELGMNPSHCNEMSWIKSIPFIHLG-KQN-LDDLLNRNNTFKPFAEYKSDYVYQPFP

Secc4.0201(CAH92630) SRFPELGMNASHCNEMPWIKSVPFIHLG-KQATLSDLLNRNNTFKPFAEYKSDYVYQPVP

Bradi1g38260.1 SKFPELGFNRGQCNEMPWAQTIPFIHLG--NRDLGDLTNRNNNFKPFAEYKSDYVYQPIP

Lolp4(Q5T1W3) KSVWEQIFGTWLVKPGAGIMIFDPYGATISATPEAATPFPHRKGVLFNIQYVNYWFA--P

Phlp4.0201(CAD54671) KTVWEQILNTWLVKPGAGIMIFDPYGATISATPESATPFPHRKGVLFNIQYVNYWFA--P

Phlp4.0101(CAD54670) KEVWEQIFSTWLLKPGAGIMIFDPYGATISATPEWATPFPHRKGVLFNIQYVNYWFA--P

Tria4.0101(CAH92632) KPVWEQIFG-WLTKPGGGMMIMDPYGATISATPEAATPFPHRQGVLFNIQYVNYWFA--E

Tria4.0201(CAH92633) KPVWEQIFG-WLAKPGAGIMIMDPYGATISATPEAATPFPHRQGVLFNIQYVNYWFA--E

Secc4.0101(CAH92627) KPVWEQIFG-WLVKPGAGIMIMDPYGATISATPEAATPFPHRQGVLFNIQYVNYWFA--E

Secc4.0201(CAH92630) KPVWAQIFV-WLVKPGAGIMVMDPYGAAISATPEAATPFPHRKDVLFNIQYVNYWFD--E

Bradi1g38260.1 KNVWEQIFG-WLTKPGAGIMIMDPYGATISATPETATPFPHRKGVLFNIQYVNYWFA—-E

Lolp4(Q5T1W3) GAGAAPLSWSKEIYNYMEPYVSKNPRQAYANYRDIDLGRNEVVNG----VSTYSSGKVWG

Phlp4.0201(CAD54671) GAAAAPLSWSKDIYNYMEPYVSKNPRQAYANYRDIDLGRNEVVND----VSTYASGKVWG

Phlp4.0101(CAD54670) GAGAAPLSWSKEIYNYMEPYVSKNPRQAYANYRDIDLGRNEVVND----VSTFSSGLVWG

Tria4.0101(CAH92632) AAAAAPLQWSKDMYNFMEPYVSKNPRQAYANYRDIDLGRNEVVND----ISTYSSGKVWG

Tria4.0201(CAH92633) PAGAAPLQWSKDIYNFMEPYVSKNPRQAYANYRDIDLGRNEVVND----ISTYSSGKVWG

Secc4.0101(CAH92627) SAGAAPLQWSKDIYKFMEPYVSKNPRQAYANYRDIDLGRNEVVND----ISTYSSGKVWG

Secc4.0201(CAH92630) AGGAAPLQWSKDMYRFMEPYVSKNPRQAYANYRDIDLGRNEVVND----ISTYASGKVWG

Bradi1g38260.1 GAGAAPLQWSKDMYKFMEPYVSKNPRQAYANYRDIDLGRNEVVND----VSTYSSGKVWG

Lolp4(Q5T1W3) QKYFKGNFERLAITKGKVDPTDYFRNE-------------------------

Phlp4.0201(CAD54671) QKYFKGNFERLAITKGKVDPTDYFRNEQSIPPLIKKY---------------

Phlp4.0101(CAD54670) QKYFKGNFQRLAITKGKVDPTDYFRNEQSIPPLIQKY---------------

Tria4.0101(CAH92632) EKYFKGNFQRLAITKGKVDPQDYFRNEQSIPPLLEKY---------------

Tria4.0201(CAH92633) EKYFKSNFQRLAITKGKVDPQDYFRNEQSIPPLIEKY---------------

Secc4.0101(CAH92627) EKYFKGNFQRLAITKGKVDPQDYFRNEQSIPPLVEKY---------------

Secc4.0201(CAH92630) EKYFKGNFQRLAITKGKVDPQDYFRNEQSIPPLLGK----------------

Bradi1g38260.1 EKYFKGNFQRLAMIKGKVDPEDYFRNEQSIPPLLTKY---------------

Light gray: conserved residues

Dark gray: Conservative substitutions

Turquoise: N-glycosylation sites
