# Supplementary material for: Comparative and Evolutionary Analysis of Grass Pollen Allergens Using Brachypodium distachyon as a Model System
Source: PLoS One. 2017 Jan 19;12(1):e0169686. doi: 10.1371/journal.pone.0169686 (PMC5245863; doi:10.1371/journal.pone.0169686)
Supplement: S8 Fig — The protein sequences were aligned by Clustal X2.0 and conserved residues were highlighted in different colors. (DOC) [file pone.0169686.s008.doc]

Phlp5 (Q40960) ------------------------------------------------------------

Lolp5 (Q40237) ------------------------------------------------------------

Lolp5 (Q40240) ------------------------------------------------------------

Holl5.0201(CAB10766) ------------------------------------------------------------

Holl5.0101(CAB10765) ------------------------------------------------------------

Dacg5(CAD20402) ------------------------------------------------------------

Dacg5(CAD20403) ------------------------------------------------------------

Dacg5(CAD20404) ------------------------------------------------------------

Dacg5(CAD20405) ------------------------------------------------------------

Phlp5 (Q40963) ------------------------------------------------------------

Poap5 (AAG42254) ------------------------------------------------------------

Phaa5 (P56164) ------------------------------------------------------------

**Bradi3g02660.1** ------------------------------------------------------------

**Bradi3g02665.1** ------------------------------------------------------------

Horv5.0101(AAB41585) --------------------------------------------------------MANS

**Bradi2g48255.1** ------------------------------------------------------------

Phlp5 (Q40960) -------------------------------ADLGYGPATPAAPAAGYTPATPAAPAE--

Lolp5 (Q40237) ----------------------------ADAGYAPATPATPAAPATAATPATPATPATPA

Lolp5 (Q40240) ----------------------------------------ADAGYTPAAAATPATPAATP

Holl5.0201(CAB10766) ------------------------------------------------------------

Holl5.0101(CAB10765) ----------------------------------------ADAGYTPAAPAAAG------

Dacg5(CAD20402) ----------------------------------------ADAGYTPAAAATPAT-----

Dacg5(CAD20403) ----------------------------------------ADAGYTPAAAATPAT-----

Dacg5(CAD20404) ----------------------------------------ADAGYTPAAAATPAT-----

Dacg5(CAD20405) ----------------------------------------ADAGYTPAAAATPAA-----

Phlp5 (Q40963) ---------------------------AAAAVPRRGPRGGPGRSYTADAGYAPATPAAAG

Poap5 (AAG42254) ----------------------------------------------ADAGYAPATPAAAG

Phaa5 (P56164) -----------------------------------------------TPPTPRTPPLLPP

**Bradi3g02660.1** ---------------------------------------------------YAPVTPATP

**Bradi3g02665.1** ---------------------------------------------------YAPVTPATP

Horv5.0101(AAB41585) GREHSAVPRRRNLVALVPRHGCYAEFSLYVCVGNINAPFPVFNRTTFIANAGIEAELEPH

**Bradi2g48255.1** ------------------------------------------------------------

Phlp5 (Q40960) AAPAGKATTEEQKLIEKINAGFKAALAAAAGVQPADKYRTFVATFGAASNKAFAEGLSGE

Lolp5 (Q40237) AVPSGKATTEEQKLIEKINAGFKAAVAAAAVVPPADKYKTFVETFGTATNKAFVEGLASG

Lolp5 (Q40240) AAAGGKATTDEQKLLEDVNAGFKAAVAAAANAPPADKFKIFEAAFSESSKGLLATS---A

Holl5.0201(CAB10766) -----------QKLLEDVNASFKAAVAAAAKVPPADKYKTFLRAFTVLDRGSTEQSKAEE

Holl5.0101(CAB10765) --AGGKATTDEQKLLEDVNAGFKTAVAAAANVPPADKYKTFEAAFTASSKASIAAA---A

Dacg5(CAD20402) --AGGKAMTEEQTLIEDVNAGFKAAVAAASSAPPADKFKTFEATFTAACKANIAAA---A

Dacg5(CAD20403) --AGGKAMTEEQTLIEDVNAGFKAAVAAASSAPPADKFKTFEATFTAACKANIAAA---A

Dacg5(CAD20404) --AGGKAMTEEQTLIEDVNAGFKAAVAAASSAPPADKFKTFEATFTAACKANIAAA---A

Dacg5(CAD20405) --AGGKAMTEEQKLIEDVNAGFKAAVAAASSAPPADKFKTFEATFTAACKANIAAA---A

Phlp5 (Q40963) AAAGKAT-TEEQKLIEDINVGFKAAVAAAASVPAADKFKTFEAAFTS-----SSKAA--A

Poap5 (AAG42254) AAAGKITPTQEQKLMEDINVGFKAAVAAAAGAPPADKFKTFQAAFSASVEASAAKLN--A

Phaa5 (P56164) PRARDKATLTSRSVEDINAASRRPWWASVP---PADKFKTFADHVLCVPNADVTSAAT--

**Bradi3g02660.1** AAGYPKASTDEQKMIEDINAGFKTAVAAANAAPPADKYNTFEAAFKKASTGYKAGVSA--

**Bradi3g02665.1** AAGYPKASTDEQKMIEDINAGFKTAVAAANAAPPADKYNTFEAAFKKASTGYKAG-----

Horv5.0101(AAB41585) FLLLLFTFSSSSSFFTLLKTMIHFTDRSDNKNKAMMRGREFRKAFAEVLKGAATGQIAGQ

**Bradi2g48255.1** ------------------------------------------------------------

Phlp5 (Q40960) PKGAAESSSKAALTSKLDAAYKLAYKTAEGATPEAKYDAYVATLSEALRIIAGTLEVHAV

Lolp5 (Q40237) ----YADQSKNQLTSKLDAALKLAYEAAQGATPEAKYDAYVATLTEALRVIAGTLEVHAV

Lolp5 (Q40240) AKAPGLIP-------KLDTAYDVAYKAAE-ATPEAKYDAFVTALTEALRVIAGALEVHAV

Holl5.0201(CAB10766) TKMPELSS-------KLVDAYMAAFKASTGGTQEAKYDAFVTTLTEALRVIAGALEVHAV

Holl5.0101(CAB10765) TKAPGLIP-------QLNAATNTAYAAAQGATPEAKYDAFVTTLTEALRVIAGALEVHAV

Dacg5(CAD20402) TKVPLFVA-------KLDAAYAVAYKTATGPTPEAKYDAFVAALTEALRVIAGALEVHAV

Dacg5(CAD20403) TKVPLFVA-------KLDAAYAVAYKTATGPTPEAKYDAFVAALTEALRVIAGALEVHAV

Dacg5(CAD20404) TKVPLFVA-------KLDAAYAVAYKTATGPTPEAKYDAFVAALTEALRVIAGALEVHAV

Dacg5(CAD20405) TKVPLFVA-------KLDAAYAVAYKTAAGPTPEAKYDAFVAALTEALRVIAGALEVHAV

Phlp5 (Q40963) AKAPGLVP-------KLDAAYSVAYKAAVGATPEAKFDSFVASLTEALRVIAGALEVHAV

Poap5 (AAG42254) AQAPGFVS-------HVAATSDATYKAAVGATPEAKFDSFVAAFTEALRIIAGVLKVHAV

Phaa5 (P56164) -KAPQLKA-------KLDAAYRVAYEAAEGSTPEAKYDAFIAALTEALRVIAGAFEVHAV

**Bradi3g02660.1** ---------------KLDTAYQAAYKAADGATPEAKYDVFVATLTEALRVVAGAVEAHAV

**Bradi3g02665.1** ----------------------AAYKAADGATPEAKYDVFVATLTEALRVVAGAVEAHAV

Horv5.0101(AAB41585) SSSMAKLSS------SLELSYKLAYDKAQGATPEAKYDAYVATLTESLRVISGTLEVHSV

**Bradi2g48255.1** ------------------------------------------------------------

Phlp5 (Q40960) KPAAEEVKV-------IPAGELQVIEKVDAAFKVAATAAN---------AAPANDKFTVF

Lolp5 (Q40237) KPAAEEVKVGA-----IPAAEVQLIDKVDAAYRTAATAAN---------AAPANDKFTVF

Lolp5 (Q40240) KPATEEVLAAK-----IPTGELQIVDKIDAAFKIAATAAN---------AAPTNDKFTVF

Holl5.0201(CAB10766) KPATEEVPAAK-----IPAGDLQVVDKIDASFKIAATAAN---------AAPANDKFTVF

Holl5.0101(CAB10765) KPATEEVGAAK-----IPAGELQIVDKIDAAFRIAATAAN---------AAPVNDKFTVF

Dacg5(CAD20402) KPAAEEVPAAK-----IPAGELQIVDKIDAAYKIAATAAN---------AAPANDKFTVF

Dacg5(CAD20403) KPAAEEVPAAK-----IPAGELQIVDKIDAAYKIAATAAN---------AAPANDKFTVF

Dacg5(CAD20404) KPAAEEVPAAK-----IPAGELQIVDKIDAAYKIAATAAN---------AAPANDKFTVF

Dacg5(CAD20405) KPAAEEVPAAK-----IPAGELQIVDKIDAAYKIAATAAN---------AAPANDKFTVF

Phlp5 (Q40963) KPVTEEPGMAK-----IPAGELQIIDKIDAAFKVAATAAA---------TAPADDKFTVF

Poap5 (AAG42254) KPITEETGAAK-----IPAGEQQIIDKIDAAFKVAATAAN---------AAPANDKFTVF

Phaa5 (P56164) KPATEEVVAD-------PVGELQIVDKIDAAFKIAATAAN---------SAPANDKFTVF

**Bradi3g02660.1** KPVAEELAAGAGGK--MPADELKIVDKIDAAFKTASTAAN---------AAPANDKFNVF

**Bradi3g02665.1** KPVAEELAAGAGGK--MPADELKIVDKIDSAFKTASTAAN---------AAPANDKFNVF

Horv5.0101(AAB41585) KPAAEEVKG-------VPAGELKAIDQVDAAFRTAATAAD---------AAPANDKFTVF

**Bradi2g48255.1** ------------------------------------------------------------

Phlp5 (Q40960) EAAFNDAIKASTGGAYESYKFIPALEAA-VKQAYAATVATAPEVKYTVFETALKKAITAM

Lolp5 (Q40237) ENTFNNAIKVSLGAAYDSYKFIPTLVAA-VKQAYAAKQATAPEVKYTVSETALKKAVTAM

Lolp5 (Q40240) ESAFNKALNECTGGAYETYKFIPSLEAA-VKQAYAATVAAAPEVKYAVFEAALTKAITAM

Holl5.0201(CAB10766) ETAFNKALKESTGGAYESYKFIPSLEAA-VKQAYASTVAAAPEVKYAVFEAALTKAITAM

Holl5.0101(CAB10765) EGAFNKAIKESTGGAYEAYKFIPSLETA-VKQAYAATVATAPEVKYTVFETALKKAITAM

Dacg5(CAD20402) EGAFNKAIKESTGGAYESYKFIPTLEAA-VKQAYAATVAAAPEVKYAVFEAALTKAITAM

Dacg5(CAD20403) EGAFNKAIKESTGGAYESYKFIPTLEAA-VKQAYAATVAAAPEVKYAVFEAALTKAITAM

Dacg5(CAD20404) EGAFNKAIKESTGGAYESYKFIPTLEAA-VKQAYAATVAAAPEVKYAVFEAALTKAITAM

Dacg5(CAD20405) EGAFNKAIKESTGGAYESYKFIPTLEAA-VKQAYAATVAAAPEVKYAVFEAALTKAITAM

Phlp5 (Q40963) EAAFNKAIKESTGGAYDTYKCIPSLEAA-VKQAYAATVAAAPQVKYAVFEAALTKAITAM

Poap5 (AAG42254) EAAFNNAIKESTGGAYDTYKSIPSLEAA-VKQAYAATIAAAPEVKFAVFKAALTKAITAM

Phaa5 (P56164) EGAFNKAIKESTAGAYETYKFIPSLEAA-VKQAYGATVARAPEVKYAVFEAGLTKAITAM

**Bradi3g02660.1** EATFNKALKESTGGAYESYKFIPSLEAA-VKQAYAATIAAAPEVKYAVFEAALTKAITAM

**Bradi3g02665.1** EATFNKALKESTGGAYESYKFIPSLEAA-VKQAYAATIAAAPEVKYAVFEAALTKAITAM

Horv5.0101(AAB41585) ESLQQGPSRKPRGGAYESYKFIPALEAA-VKQAYAATVAAAPEVKFTVFQTALSKAINAM

**Bradi2g48255.1** -----MAGMSIDGKAYTDYKFIPPLDAA-FKEAYASTVAAKPEVKYAVFEAALTKAIGAM

Phlp5 (Q40960) SEAQKAAKPAAAATATATAAVGAATGAATAATGGYKV-----------------------

Lolp5 (Q40237) SEAEKEATPAAAATATPTPAAATATATPAAAYATATPAAATATATPAAATATPAAAGGYK

Lolp5 (Q40240) TQAQKAGKPAAAAATAAATVATAAATAAAVLPPPLLVVQSLI------------------

Holl5.0201(CAB10766) SQAQKVAQPAAAATGAATVAAGAATTAAGGYKV---------------------------

Holl5.0101(CAB10765) SEAQKEAKPVAAATGAATAAAGVAAGAATAAAGGYKV-----------------------

Dacg5(CAD20402) SEAQKVATPAAVATGAATAAASAATGAATAAAGGYKV-----------------------

Dacg5(CAD20403) SEAQKVATPAAVATGAATAAASAATGAATAAAGGYKV-----------------------

Dacg5(CAD20404) SEAQKVATPAAVATGAATAAASAATGAATAAAGGYKV-----------------------

Dacg5(CAD20405) SEAQKVATPAAAATGAATAAASAATGAATAAAGGYKV-----------------------

Phlp5 (Q40963) SEVQKVSQPATGAATVAAGAATTAAGAAS-------GAATVA------------------

Poap5 (AAG42254) AEVQKVSKPVAGAATVAAGAATAATGAATGAAGAATGAATVS------------------

Phaa5 (P56164) SEAQKVAKPP-LSPQPPQVLPLAAGGAATVAAASDVRVCRSHGTLQDACLLRCRGGCQPV

**Bradi3g02660.1** SEAQKAKGGVTVTATVSAGAAAKPAAPGYKFYIYSLILIYHFY-----------------

**Bradi3g02665.1** SEAQKAKGAAGYAGAAAGAGATVAAGAGAAVSAGGYKV----------------------

Horv5.0101(AAB41585) TQAGKVAKPAAAATATATVAAGAAATAGNYKV----------------------------

**Bradi2g48255.1** AEAEKAASTKPAA-----------------------------------------------

Phlp5 (Q40960) ---------------

Lolp5 (Q40237) V--------------

Lolp5 (Q40240) ---------SLLIYY

Holl5.0201(CAB10766) ---------------

Holl5.0101(CAB10765) ---------------

Dacg5(CAD20402) ---------------

Dacg5(CAD20403) ---------------

Dacg5(CAD20404) ---------------

Dacg5(CAD20405) ---------------

Phlp5 (Q40963) ---------AGGYKV

Poap5 (AAG42254) ---------AGGYKV

Phaa5 (P56164) VWRGGSHRARGGYKV

**Bradi3g02660.1** ---------------

**Bradi3g02665.1** ---------------

Horv5.0101(AAB41585) ---------------

**Bradi2g48255.1** ---------------

Light gray: conserved residues

Dark gray: Conservative substitutions

Red: IgE-binding residues
