# Supplementary material for: Comparative and Evolutionary Analysis of Grass Pollen Allergens Using Brachypodium distachyon as a Model System
Source: PLoS One. 2017 Jan 19;12(1):e0169686. doi: 10.1371/journal.pone.0169686 (PMC5245863; doi:10.1371/journal.pone.0169686)
Supplement: S10 Fig — The protein sequences were aligned by Clustal X2.0 and conserved residues were highlighted in different colors. (DOC) [file pone.0169686.s010.doc]

Phlp7 (O82040) --MADDMERIFKRFDTNGDGKISLSELTDALRTLGSTSADEVQRMMAEIDTDGDGFIDFN

Bradi4g21210.1 MAAAEDMERIFKRFDTNGDGKISLSELTDALRTLGSTSADEVQRMMAEIDTDGDGFIDFD

Cynd7 (P94092) MADTGDMEHIFKRFDTNGDGKISLAELTDALRTLGSTSADEVQRMMAEIDTDGDGFIDFD

Phlp7 (O82040) EFISFCNANPGLMKDVAKVF

Bradi4g21210.1 EFISFCNANPGLMKDVAKVF

Cynd7 (P94092) EFISFCNANPGLMKDVAKVF

Yellow: Two Calcium-binding domains (EF-hands)

Gray: Conserved residues
