# Supplementary material for: Comparative and Evolutionary Analysis of Grass Pollen Allergens Using Brachypodium distachyon as a Model System
Source: PLoS One. 2017 Jan 19;12(1):e0169686. doi: 10.1371/journal.pone.0169686 (PMC5245863; doi:10.1371/journal.pone.0169686)
Supplement: S12 Fig — The protein sequences were aligned by Clustal X2.0 and conserved residues were highlighted in different colors. (DOC) [file pone.0169686.s012.doc]

**Bradi2g31950.1** QGYSKPGFLVTGRVYCDTCRAGFETNASHSIPGAVVQMECRHFETNELHHKAEATTDAQG

**Bradi2g31950.2** QGYSKPGFLVTGRVYCDTCRAGFETNASHSIPGAVVQMECRHFETNELHHKAEATTDAQG

Lolp11(Q7M1X5) --DKGPGFVVTGRVYCDPCRAGFETNVSHNVEGATVAVDCRPFDGGESKLKAEATTDKDG

Phlp11(AAN32987) --DKGPGFVVTGRVYCDPCRAGFETNVSHNVQGATVAVDCRPFNGGESKLKAEATTDGLG

**Bradi2g07610.1** YEKGPGGFVVTGRVYCDPCRAGFETNVSKNIGGATVAVDCRPFNGGDSKLKAEATTDQYG

**Bradi5g08410.1** --VDLPDYIVQGRVYCDTCRAGFETNVTTYIKGAKVRLECKRFGTEKVERALDGVTDETG

**Bradi2g31950.1** WYKMEVGEDHQEEICEVALLRSPEKDCAEIEKSRDRCRVPLTRNNGIKQSGVRYANPIAF

**Bradi2g31950.2**  WYKMEVGEDHQEEICEVALLRSPEKDCAEIEKSRDRCRVPLTRNNGIKQSGVRYANPIAF

Lolp11(Q7M1X5) WYKIEIDQDHQEEICEVVLAKSPDKSCSEIEEFRDRARVPLTSNXGIKQQGIRYANPIAF

Phlp11(AAN32987) WYKIEIDQDHQEEICEVVLAKSPDTTCSEIEEFRDRARVPLTSNNGIKQQGIRYANPIAF

**Bradi2g07610.1** WYKIDIDQDHQEEICEVLLARSPDPACSEIEEFRDRARVPLTRNNGLKQQGTRYANPIAF

**Bradi5g08410.1** TYKIELKDSHPEDICEVVLIQSPLADCNKIQALRDRARVELTRNIGISDN-LRLANSLGY

**Bradi2g31950.1** FRKEPLANCGDVLRKYDLYDETSENS---

**Bradi2g31950.2** FRKEPLANCGDVLRKYDLYDETSENS---

Lolp11(Q7M1X5) FRKEPLKECGGILQAY-------------

Phlp11(AAN32987) FRKEPLKECGGILQAYDLRDAPETP----

**Bradi2g07610.1** FRKEPLKDCGGILQAYDLKDAPETP----

**Bradi5g08410.1** LKDVPLPVCAQLLKQFKSADDDDDDQVKA

Green: Conserved cysteine residues

Turquoise: N-glycosylation sites

Light gray: conserved residues

Dark gray: Conservative substitutions
