# Supplementary material for: Comparative and Evolutionary Analysis of Grass Pollen Allergens Using Brachypodium distachyon as a Model System
Source: PLoS One. 2017 Jan 19;12(1):e0169686. doi: 10.1371/journal.pone.0169686 (PMC5245863; doi:10.1371/journal.pone.0169686)
Supplement: S14 Fig — The protein sequences were aligned by Clustal X2.0 and conserved residues were highlighted in different colors. (DOC) [file pone.0169686.s014.doc]

Zeam12 (P35081) MSWQTYVDEHLMCEIEGHHLTSAAIVGHDGATWAQSTAFPEFKPEEMAAIMKDFDEPGHL

Zeam12 (P35082) MSWQAYVDEHLMCEIEGHHLAAAAIVGHDGAAWAQSTAFPEFKTEDMANIMKDFDEPGHL

Cynd12 (O04725) MSWQAYVDDHLMCEIEGHHLTSAAIIGHDGTVWAQSAAFPAFKPEEMANIMKDFDEPGFL

Orys12 (AAG32056) MSWQTYVDEHLMCEIEGHHLTSAAIVGHDGTVWAQSAAFPQFKPEEMTNIMKDFDEPGFL

Zeam12 (P35083) MSWQTYVDEHLMCEIEGHHLSSAAIVGHDGAVWAQSTAFPQFKPEEMTNIIKDFDEPGFL

Phlp12 (P35079) MSWQTYVDEHLMCEIEGHHLASAAILGHDGTVWAQSADFPQFKPEEITGIMKDFDEPGHL

**Bradi2g49340.1** MSWQTYVDEHLMCDIEGHHLVSAAILGHDGTVWAQSADFPSFKPEEMTNIMKDFDEPGTL

**Bradi2g19360.1** MSWQTYVDEHLMCEIEGHHLGSAAILGHDGTVWAQSAAFPAFEPKEMTDIMKDFDEPGHL

Zeam12 (P35081) APTGLILGGTKYMVIQGEPGAVIRGKKGSGGITVKKTGQSLIIGIYDEPMTPGQCNLVVE

Zeam12 (P35082) APTGLFLGPTKYMVIQGEPGAVIRGKKGSGGITVKKTGQALVVGIYDEPMTPGQCNMVVE

Cynd12 (O04725) APTGLFLGPTKYMVIQGEPGAVIRGKKGSGGVTVKKTGQALVIGIYDEPMTPGQCNMVIE

Orys12 (AAG32056) APTGLFLGPTKYMVIQGEPGAVIRGKKGSGGITVKKTGQALVVGIYDEPMTPGQCNMVVE

Zeam12 (P35083) APIGLFLGPTKYMVIQGEPGAVIRGKKGSGGITVKKTGQALVIGIYDEPMTPGQCNMVVE

Phlp12 (P35079) APTGMFVAGAKYMVIQGEPGRVIRGKKGAGGITIKKTGQALVVGIYDEPMTPGQCNMVVE

**Bradi2g49340.1** APTGLFLASAKYMVIQGEPGAVIRGKKGSGGITLKKTGQALVVGIYDEPMTPGQCNMVVE

**Bradi2g19360.1** APTGMFLGGAKYMVIAGEPGAVIRGKKGSGGITIKKTGQALVIGIYDEPMTPGQCNMVVE

Zeam12 (P35081) RLGDYLLEQGM

Zeam12 (P35082) RLGDYLLEQGM

Cynd12 (O04725) KLGDYLIEQGM

Orys12 (AAG32056) RLGDYLVEQGL

Zeam12 (P35083) RLGDYLVEQGL

Phlp12 (P35079) RLGDYLVEQGM

**Bradi2g49340.1** RLGDYLVEQGM

**Bradi2g19360.1** RLGDYLVEQGM

Yellow: Conserved cysteine residues

Light Gray: conserved residues

Dark Gray: conserved substitutions
