# Supplementary material for: Comparative and Evolutionary Analysis of Grass Pollen Allergens Using Brachypodium distachyon as a Model System
Source: PLoS One. 2017 Jan 19;12(1):e0169686. doi: 10.1371/journal.pone.0169686 (PMC5245863; doi:10.1371/journal.pone.0169686)
Supplement: S16 Fig — The protein sequences were aligned by Clustal X2.0 and conserved residues were highlighted in different colors. (DOC) [file pone.0169686.s016.doc]

**Bradi1g36110.1** ------KAAEKEAAGSAAAASGPGATLDIVKLG---------ATGDGKTDSTKAVEEAWA

**Bradi1g36090.1** ------KAAEKEAAGSAAAASGPGATLDIVKLG---------ATGDGKTDSTKAVEEAWA

Phlp13 (CAB42886) ---GKKEEKKEEKKESGDAASGADGTYDITKLG---------AKPDGKTDCTKEVEEAWA

Zeam13 (89892725) --------EKAKSKDNDAKASGPGGSFDITKLG---------ASGNGKTDSTKAVQEAWA

**Bradi1g40990.1** --EKKEKKEEKGGKDKEADAEGPAAEGPSSGGGELDISKSGKCKGDGKADCTEALEEAWA

**Bradi2g13740.1** KRPVAKESAGPAPAPAPEEASGDGGTFDITKLG---------AASDGKTDCSKELEEAWK

**Bradi3g07120.1** -----------TYAAAGGPPSVPAGPLDIVLLG---------AKGDGKTDATEAVQKAWK

**Bradi1g36110.1** SACGGTGKQTILIPKGDFLTGPLNFTGPCTG-DITIQVDGNLLGSNDLAKYKAN--WIEI

**Bradi1g36090.1** SACGGTGKQTILIPKGDFLTGPLNFTGPCTG-DITIQVDGNLLGSNDLAKYKAN--WIEI

Phlp13 (CAB42886) SACGGTGKNTIVIPKGDFLTGPLNFTGPCKGDSVTIKLDGNLLSSNDLAKYKAN--WIEI

Zeam13 (89892725) SACGGTGKQTILIPKGDFLVGPLNFTGPCKG-DVTIQVNGNLLATTDLSQYKDHGNWIEI

**Bradi1g40990.1** SACKGTGKQTIQIPKGDYLTGPLNFTGPCTG-DVTIQLDGNLLGSTDMALYKSN--WIEI

**Bradi2g13740.1** SACGAAGQHTVVIPKGEFLCGPVNFSGPCEG-DVTIQIDGTLLATNDLPKYGGG-SWINI

**Bradi3g07120.1** NACAATGVQKIIIPPGNFLIGALALEGPCTS-SIIIRLDGNLLGTGDLNAYKKN--WIEV

**Bradi1g36110.1** MRVKNLVITGK-GKLDGQGPAVWGKNSCAKNYNCKILPNTLVLDFCNNTLIEGITLLNSK

**Bradi1g36090.1** MRVKNLVITGK-GKLDGQGPAVWGKNSCAKNYNCKILPNTLVLDFCNNTLIEGITLLNSK

Phlp13 (CAB42886) MRIKKLTITGK-GTLDGQGKAVWGKNSCAKNYNCKILPNTLVLDFCDDALIEGITLLNAK

Zeam13 (89892725) LRVDNLVITGK-GKLDGQGPAVWSKNSCVKKYDCKILPNSLVMDFVNNGEVSGITLLNSK

**Bradi1g40990.1** MRVENLVISGK-GTLDGQGPKVWSKNACAKKYDCKILPNSLVLDFITNGTISGITLLNAK

**Bradi2g13740.1** LKVDNLVITGS-GTLDGQGSNVWTKDPQAKAG----YPNTLVLDYVKNGSVSGITLLNSK

**Bradi3g07120.1** MHVDNFAINGH-GTIDGQGPLVWQKNQCNKNYNCKILPNSLVLDYVTNASIRGVTLKNAK

**Bradi1g36110.1** FFHLNIYECRGITVKDVTITSPGDSPNTDGIHMGDASNITITDTKIGTGDDCISIGPGTS

**Bradi1g36090.1** FFHLNIYECRGITVKDVTITSPGDSPNTDGIHMGDASNITITDTKIGTGDDCISIGPGTS

Phlp13 (CAB42886) FFHMNIYECKGVTVKDVTITAPGDSPNTDGIHIGDSSKVTITDTTIGTGDDCISIGPGST

Zeam13 (89892725) FFHMNMYKCKDMLIKDVNVTAPGDSPNTDGIHMGDSSGVTITNTVIGVGDDCISIGPGTS

**Bradi1g40990.1** FFHMNVFQCKGVTIEDVTVTAPGDSPNTDGIHIGDSSGITITGTTIGVGDDCISIGPGST

**Bradi2g13740.1** FFHMNIYMSSDVKIDNVTITAPGDSPNTDGIHIGDSSNIHVTGATIGTGDDCISIGGGSA

**Bradi3g07120.1** FFHMNLFNCKNVVVDKVNITAPGDSPNTDGIHMGDSDNITITNTNIGVGDDCISIGPGTQ

**Bradi1g36110.1** GVNISGVNCGPGHGISVGSLGRYKDEKDVTDITVKNCVLNKSTNGVRIKSYEDAKSPLVA

**Bradi1g36090.1** GVNISGVNCGPGHGISVGSLGRYKDEKDVTDITVKNCVLNKSTNGVRIKSYEDAKSPLVA

Phlp13 (CAB42886) GLNITGVTCGPGHGISVGSLGRYKDEKDVTDITVKNCVLKKSTNGLRIKSYEDAKSPLTA

Zeam13 (89892725) KVNITGVTCGPGHGISIGSLGRYKDEKDVTDINVKDCTLKKTANGVRIKAYEDAASVLTA

**Bradi1g40990.1** KINITGVTCGPGHGISIGSLGRYKDEKDVTDINVKDCTLKKATNGLRIKSYQSAESSLTA

**Bradi2g13740.1** SITVTGVTCGPGQGISVGCLGRYKDEKDVSDVTVKDCVLRSSTNGVRIKTYVDAVKSITA

**Bradi3g07120.1** RVRVHGSRCGPGHGISVGSLGRYKDEKNVEDIQVTNCTIKGATNGLRIKSYEDSKSVLKA

**Bradi1g36110.1**  SKLTYENIKMMDVGYPIIIDQKYCPNKICPPKPNSAKVTVKDVTFRNITGTSSTPEAVSL

**Bradi1g36090.1** SKLTYENIKMMDVGYPIIIDQKYCPNKICPPKPNSAKVTVKDVTFRNITGTSSTPEAVSL

Phlp13 (CAB42886) SKLTYENVKMEDVGYPIIIDQKYCPNKICTSKGDSARVTVKDVTFRNITGTSSTPEAVSL

Zeam13 (89892725) SKIHYENIKMEDSGYPIIIDMKYCPNKLCTANG-ASKVTVKDVTFKNITGTSSTPEAVNL

**Bradi1g40990.1** SEIHYENVNMEDVANPIIIDMNYCPNKICPAKG-SSHVTIKDVTFKNITGTSSTPEAVSL

**Bradi2g13740.1** SNLTFENIKMEDVANPIIIDQNYCPEKICTAKS-KSAVTVKDIIFRNITGTSSTPEAVSL

**Bradi3g07120.1** SRFVYDQIVMDNVSFPIVIDQKYCPNNICNKNG-QSTVTIQDIVFKNIVGTSATPEAVTL

**Bradi1g36110.1** LCSDKQPCSGVQMFDVKVEYSGT-NNKTMAVCTNAKVTAKGCSEALACAA--

**Bradi1g36090.1** LCSDKQPCSGVQMFDVKVEYSGT-NNKTMAVCTNAKVTAKGCSEALACAA--

Phlp13 (CAB42886) LCSDKQPCNGVTMNDVKIEYSGT-NNKTMAVCTNAKVTAKGVSEANTCAA--

Zeam13 (89892725) LCSAKIPCTGVTMDDVNIKYSGT-NNKTMAVCKNAKGSAKGCLKELACF---

**Bradi1g40990.1**  LCSDKLPCSGVELNDVKVEYSGK-NNKTMAVCKNAKGTAKGCLEALACL---

**Bradi2g13740.1** LCSEKQPCSGVELIDVNVEYSGK-NNKTMGVCTNAKGIAKDTLQALACVSDV

**Bradi3g07120.1** NCPNNLPCQGVQLVNVNLKYVGARNNKTMAVCHNAVGKSTNVAKELACL---

Yellow: Conserved polygalacturonase domains

Light Gray: conserved residues

Dark Gray: conserved substitutions

Green: cysteine residues (14 conserved) Red: N-glycosylation sites
